# Supplementary material for: Quantifying the association between psychological distress and low back pain in urban Europe: a secondary analysis of a large cross-sectional study
Source: BMJ Open. 2023 Feb 16;13(2):e047103. doi: 10.1136/bmjopen-2020-047103 (PMC9936285; doi:10.1136/bmjopen-2020-047103)
Supplement: Supplementary data [file bmjopen-2020-047103supp001.pdf]

Table A Supplementary Material

Prevalence of LBP in the EURO URHIS 2 UAs with 95% CIs

		Experienced LBP in the past one month			
Urban Area	N	%	95% CI		
			Lower CI	Upper CI	
Skopje	764	31.3	28.01	34.59	Macedonia
Birmingham	291	31.6	26.2	36.94	United Kingdom
Stockport	534	32.2	28.24	36.16	United Kingdom
Oslo	809	33.4	30.15	36.65	Norway
Utrecht	731	34.1	30.66	37.54	The Netherlands
Wirral	395	34.4	29.72	39.08	United Kingdom
Salford	399	35.6	30.9	40.3	United Kingdom
Amsterdam	663	35.7	32.05	39.35	The Netherlands
Cardiff	437	35.7	31.21	40.19	United Kingdom
Halton & St. Helens	384	36.5	31.68	41.32	United Kingdom
Bury	443	36.8	32.31	41.29	United Kingdom
Liverpool	440	37.5	32.98	42.02	United Kingdom
Tameside and Glossop	472	37.9	33.52	42.28	United Kingdom
Sefton	557	37.9	33.87	41.93	United Kingdom
Craiova	848	38	34.73	41.27	Romania
Oldham	437	38.9	34.33	43.47	United Kingdom
Tetovo	693	39.2	35.57	42.83	Macedonia
Glasgow	270	40.7	34.84	46.56	United Kingdom
Bordeaux	443	41.1	36.52	45.68	France
Montpellier	446	43	38.41	47.59	France
Izmir	136	46.3	37.92	54.68	Turkey
Knowsley	412	46.6	41.78	51.42	United Kingdom
Ankara	180	48.3	41	55.6	Turkey
Kosice	689	48.8	45.07	52.53	Slovakia
Bistrita	926	51.9	48.68	55.12	Romania
Bratislava	547	53.9	49.72	58.08	Slovakia
Oberhausen	514	54.1	49.79	58.41	Germany
Ljubljana	775	55.6	52.1	59.1	Slovenia
Iasi	751	55.8	52.25	59.35	Romania
Maribor	813	64.3	61.01	67.59	Slovenia
Kaunas	691	67.6	64.11	71.09	Lithuania
Siauliai	685	67.7	64.2	71.2	Lithuania

Table B Supplementary Material

Adult Male/Female Prevalence of Low back pain in the EURO URHIS 2 Study.

Country			Total	Low back pain<br>Percentage with LBP	95% CI<br>Lower CI	 Upper CI	P value<br>p
UK	Sex	Male	2666	34.7	32.89	36.51	.0001
		Female	2805	39.3	37.49	41.11	
Netherlands		Male	654	30.4	26.87	33.93	.001
		Female	740	38.8	35.29	42.31	
Turkey		Male	179	39.7	32.53	46.87	.001
		Female	137	57.7	49.43	65.97	
Romania		Male	1137	41.6	38.74	44.46	.0001
		Female	1388	54.0	51.38	56.62	
France		Male	429	39.6	34.97	44.23	.154
		Female	460	44.3	39.76	48.84	
Slovakia		Male	598	47.8	43.80	51.80	.028
		Female	638	54.1	50.23	57.97	
Lithuania		Male	595	65.0	61.97	68.83	.07
		Female	781	69.7	66.48	72.92	
Slovenia		Male	785	58.3	54.85	61.75	.164
		female	803	61.8	58.44	65.16	
Germany		Male	233	50.6	44.18	57.02	.154
		female	281	56.9	51.11	62.69	
Norway		Male	414	30.2	25.78	34.62	.049
		Female	395	36.7	31.95	41.45	
Macedonia		Male	1084	35.3	32.46	38.14	.723
		Female	373	34.3	29.48	39.12	
Europe Total		Male	8774	41.0	39.97	42.03	.0001
		Female	8801	48.2	47.16	49.24	
**Total**			17575	44.6	43.87	45.33	

Table C Supplementary Material

Adult age prevalence of LBP in the EURO URHIS 2 Study

			Experienced LBP in the past month			
			%	95% CI		P
	Age	N		Lower CI	Upper CI	
Lithuania	18-39	301	56.5	50.90	62.10	.0001
	40-59	396	68.4	63.82	72.98	
	60+	679	72.2	68.83	75.57	
Slovenia	18-39	298	46.6	40.94	52.26	.0001
	40-59	429	60.6	55.98	65.22	
	60+	861	64.5	61.30	67.77	
Germany	18-39	79	41.8	30.92	52.68	.039
	40-59	138	59.4	51.21	67.59	
	60+	297	54.9	49.24	60.56	
Slovakia	18-39	198	33.3	26.74	39.86	.0001
	40-59	313	48.2	42.66	53.74	
	60+	725	57.1	53.50	60.70	
Romania	18-39	877	32.3	29.21	35.39	.0001
	40-59	953	51.5	48.33	54.67	
	60+	695	64.5	60.94	68.06	
Turkey	18-39	88	47.7	37.26	58.14	.936
	40-59	140	46.4	38.14	54.66	
	60+	88	48.9	38.46	59.34	
France	18-39	114	33.3	24.65	41.95	.022
	40-59	294	47.6	41.89	53.31	
	60+	481	40.7	36.31	45.09	
UK	18-39	855	31.0	27.90	34.10	.0001
	40-59	1372	37.9	35.33	40.47	
	60+	3244	38.3	36.63	39.97	
Macedonia	18-39	680	30.0	26.56	33.44	.0001
	40-59	351	28.5	23.78	33.22	
	60+	426	48.6	43.85	53.35	
Netherlands	18-39	341	29.9	25.04	34.76	.081
	40-59	268	35.4	29.67	41.13	
	60+	785	36.8	33.43	40.17	
Norway	18-39	191	30.4	23.88	36.92	.452
	40-59	154	31.8	24.44	39.16	
	60+	464	35.1	30.76	39.44	
