# Supplementary material for: Quantifying the association between psychological distress and low back pain in urban Europe: a secondary analysis of a large cross-sectional study
Source: BMJ Open. 2023 Feb 16;13(2):e047103. doi: 10.1136/bmjopen-2020-047103 (PMC9936285; doi:10.1136/bmjopen-2020-047103)
Supplement: Author's manuscript [file bmjopen-2020-047103.draft_revisions.pdf]

BMJ Open is committed to open peer review. As part of this commitment we make the peer review history of every article we publish publicly available.

When an article is published we post the peer reviewers' comments and the authors' responses online. We also post the versions of the paper that were used during peer review. These are the versions that the peer review comments apply to.

The versions of the paper that follow are the versions that were submitted during the peer review process. They are not the versions of record or the final published versions. They should not be cited or distributed as the published version of this manuscript.

BMJ Open is an open access journal and the full, final, typeset and author-corrected version of record of the manuscript is available on our site with no access controls, subscription charges or pay-per-view fees (http://bmjopen.bmj.com).

If you have any questions on BMJ Open's open peer review process please email info.bmjopen@bmj.com

The associated mental and physical health burden of low back pain in Urban Europe: A secondary analysis of the EURO URHIS 2 Study.

Journal:	*BMJ Open*
Manuscript ID	bmjopen-2020-047103
Article Type:	Original research
Date Submitted by the Author:	23-Nov-2020

Complete List of Authors:	Ugwu, Chukwuebuka; World Health Organization Country Office for Nigeria, Communicable and Non-Communicable Diseases Cluster<br>Pope, Daniel; University of Liverpool, Department of Public Health

Keywords:	EPIDEMIOLOGY, PUBLIC HEALTH, MENTAL HEALTH

SCHOLARONE™
Manuscripts

I, the Submitting Author has the right to grant and does grant on behalf of all authors of the Work (as defined in the below author licence), an exclusive licence and/or a non-exclusive licence for contributions from authors who are: i) UK Crown employees; ii) where BMJ has agreed a CC-BY licence shall apply, and/or iii) in accordance with the terms applicable for US Federal Government officers or employees acting as part of their official duties; on a worldwide, perpetual, irrevocable, royalty-free basis to BMJ Publishing Group Ltd ("BMJ") its licensees and where the relevant Journal is co-owned by BMJ to the co-owners of the Journal, to publish the Work in this journal and any other BMJ products and to exploit all rights, as set out in our licence.

The Submitting Author accepts and understands that any supply made under these terms is made by BMJ to the Submitting Author unless you are acting as an employee on behalf of your employer or a postgraduate student of an affiliated institution which is paying any applicable article publishing charge ("APC") for Open Access articles. Where the Submitting Author wishes to make the Work available on an Open Access basis (and intends to pay the relevant APC), the terms of reuse of such Open Access shall be governed by a Creative Commons licence – details of these licences and which Creative Commons licence will apply to this Work are set out in our licence referred to above.

Other than as permitted in any relevant BMJ Author's Self Archiving Policies, I confirm this Work has not been accepted for publication elsewhere, is not being considered for publication elsewhere and does not duplicate material already published. I confirm all authors consent to publication of this Work and authorise the granting of this licence.

For peer review only

TITLE PAGE

TITLE: The associated mental and physical health burden of low back pain in Urban Europe: A secondary analysis of the EURO URHIS 2 Study.

CORRESPONDING AUTHOR:

Chukwuebuka Immanuel UGWU (MBBS, MPH)

WHO Field Office, Jerome Udoji Secretariat, Awka Anambra State, Nigeria.

Email: mazibukaz@gmail.com

CO-AUTHOR:

Daniel Pope (BSc. (hons) MSc., PhD)

Professor of Global Public Health and Epidemiology, Department of Public Health and Policy, Institute of Population Health Sciences, University of Liverpool, UK.

Email: danpope@liverpool.ac.uk

WORD COUNT: 3,206

For peer review only - http://bmjopen.bmj.com/site/about/guidelines.xhtml

ABSTRACT

Objectives.

This study aims to estimate the prevalence of low back pain in Europe and to quantify its associated mental and physical health burdens among adults in European urban areas.

Design.

This research is a secondary analysis of data from a large multi-country population survey.

Setting.

The population survey on which this analysis is based was conducted in 32 European urban areas across 11 countries.

Participants.

The dataset for this study was collected during the European Urban Health Indicators System 2 survey. There were a total of 19, 441 adult respondents but data from 18,028, 50.2% female (9,050) and 49.8% Male (8,978) were included in these analyses.

Primary and secondary outcome measures

Being a survey, data on the exposure (low back pain) and outcomes were collected simultaneously. The primary outcomes for this study are psychological distress and poor physical health.

Results.

The European Prevalence of low back pain was 44.6% (43.9-45.3) widely ranging from 33.4% in Norway to 67.7% in Lithuania. The female sex, 60+ age group, lower socio-economic group, and

For peer review only

For peer review only - http://bmjopen.bmj.com/site/about/guidelines.xhtml

people with primary education were at highest risk with odds ratios of 1.34 (1.26-1.42), 1.74 (1.61-1.88), 1.96 (1.83-2.11), and 2.28 (2.05-2.54) respectively.

It was also associated with psychological distress and poor self-rated health with adjusted odds ratios of 1.44 (1.32-1.58), and 3.54 (3.31-3.40) respectively. This association varied widely between countries and cities.

Conclusion.

Prevalence of low back pain, and its associations with poor physical and mental health, vary widely and differently across European urban areas.

KEY WORDS

Low back pain, self-rated health, psychological distress, urban Europe,

STRENGTHS AND LIMITATIONS OF THE STUDY

- A key strength of this study is that it is based on uniformly collected data from a large population survey (with over 19,000 respondents) across 32 European urban areas in 11 countries and thus the parameters measured and reported are comparable across cities and countries.
- Despite measures taken to minimise the risk of bias, we acknowledge that this study may be at risk of information bias and selection bias by non-response as it is based on data from a cross-sectional survey.
- This study specifically focuses on urban areas in Europe and a standardised definition of 'urban area' was utilised in the survey to avoid misclassification.

For peer review only - http://bmjopen.bmj.com/site/about/guidelines.xhtml

For peer review only

Summary box

What is already known on his subject?

It is well known that low back pain is a leading cause of disability globally. Its prevalence in Europe is also known to vary from country to country although methodological issues greatly limit comparisons across settings. Also, the older age group, the less educated, and persons of lower socio-economic status tend to suffer more from the disease.

What does this study add?

This study specifically describes the distribution of low back pain and estimates its associated mental health burden across 32 European cities (11 countries) drawing from a large uniformly collected dataset. Notably also, we found that its associated mental health effect varies differently from the prevalence such that urban areas and countries with comparatively lower prevalence of low back pain may have much higher associated mental health burden.

INTRODUCTION

In the past few decades, low back pain (LBP) has become increasingly recognised as a huge public health concern whose impact is expected to grow as life expectancy continues to improve around the world. At present, it is a leading cause of disability [1] and ranks among the top ten causes of overall disease burden globally.[2] LBP represents a substantial burden to both society and the individual. The costs to society include associated utilisation of primary care and other health services for prolonged treatments, complex physical and surgical interventions, resulting in enormous financial costs to health services.[3,4] LBP is also a major reason for temporary or

For peer review only - http://bmjopen.bmj.com/site/about/guidelines.xhtml

permanent shortages in the workforce through sickness absence with sufferers requiring disability benefits, having a direct negative effect on the economy.[5]

In terms of burden to individuals, there is an increased need for supportive care and reduced incomes following worklessness, LBP can thus constitute a strain on family resources.[6] Also, it significantly interferes with a person's ability to engage in meaningful active social life predisposing sufferers to further morbidity and reducing individuals' overall quality of life.[7] Despite increasing treatment options such as pharmaceutical options, physical therapy, spinal manipulation and surgery,[8] outcomes are usually disappointing.[9]

Psychological distress (PD) is a known mental health problem quite common in modern populations. It has been shown to play a key role in both the aetiologic and prognostic courses of LBP in adults.[10-12] PD impacts greatly on response to LBP therapy, [10] development of disability,[11] and chronicity[13] of LBP all of which have significant economic implications.[5] Conversely, background PD is a recognised predictor of LBP in adults.[14]

Hitherto, however, there has not been any robust cross-national study exploring PD associated with LBP. Previous attempts had limited comparability across national borders due to the heterogeneity of definitions and data collection tools, differing baseline and outcome measures as well as issues with study design,[15] limiting their usefulness. This study aims to use a large, standardised, uniformly collected dataset to estimate the burden of low back pain across Europe as well as its associated mental health burden. In addition, although LBP affects both urban and rural populations, the burden is expected to differ across the two settings.[1] This is because urbanisation is associated with lifestyle changes that affect disease burden[16] and like LBP, urbanisation trend is expected to rise in the coming decades.[17] Again, no study has specifically explored PD associated LBP among urban dwellers.

For peer review only - http://bmjopen.bmj.com/site/about/guidelines.xhtml

METHODS

Overview of EURO URHIS 2 Survey setting.

This research is based on data from the second European Urban Health Indicator System Survey (EURO-URHIS 2) conducted in 2011. The initiative was developed in an attempt to build an urban health indicator system that will help to understand the health of urban populations across Europe.[18] The project set out to develop and utilise standardised health survey tools and methodologies which would be applicable to heterogeneous Urban Areas (UAs) within the region. A key objective was to ensure the collection of reliably comparable data across UAs on five key domains of urban health and determinants of health which include: demography; lifestyle; health status; health service utilisation and environmental health.[18]

32 UAs across 11 countries participated in the study[18] France (Bordeaux, Montpellier), Germany (Oberhausen), Lithuania (Kaunas, Siauliai), Macedonia (Skopje, Tetovo), Norway (Oslo), Romania (Bistrita, Craiova, Iasi), United Kingdom (Birmingham, Cardiff, Glasgow, Greater Manchester – 5 UAs, Merseyside – 5 UAs), Slovenia (Ljubljana, Bratislava, Maribor, Kosice), Turkey (Ankara, Izmir), The Netherlands (Amsterdam, Utrecht). The EURO-URHIS 2 project clearly defined comparable boundaries applicable to participating UAs avoiding the problem of mis-classifying sparsely populated urban areas and densely populated villages. [19] Standard population-based survey methods were used in the adults' research on which this work is based. Representative random samples stratified by age (18 – 64; ≥65) and sex (male; female) were obtained from available population registers in each UA. Other methodological details of the EURO-URHIS 2 including the validation processes of data collection tools and the multi-lingual translations have been published elsewhere.[18]

For peer review only - http://bmjopen.bmj.com/site/about/guidelines.xhtml

Measurement of key variables.

The one-month period prevalence of low back pain (LBP) was assessed using a pre-validated question on back pain.[20] Individuals were asked: 'In the past month have you had low back pain which lasted one day or longer?' with the option of indicating 'yes' or 'no'. The dependent variable Psychological Distress (PD) was measured using the 12 – item General Health Questionnaire (GHQ).[21] The GHQ is a validated population-based screening tool for PD.[22] It is considered to be the gold standard in assessing PD and thus has been widely used in LBP[23,24] and non-LBP surveys.[22] Individuals were required to answer each of the 12 questions on the GHQ using a four-point severity scale to assess PD. Before analysing of the PD variable, the four possible responses were dichotomised into negative (scores 1 and 2) and positive (scores 3 and 4) according to severity. So on each item of the GHQ, a respondent will then have either 'negative' or 'positive'. Thereafter, an overall PD score was computed for each person with a lowest of zero and a highest possible score of 12. Overall PD score equal or greater than 4 was considered significant to yield a binary outcome with scores 0 – 3 'No PD' and scores 4 – 12 'PD'.

The state of physical health was assessed with a 'Likert-type scale' self-rated health question in which participants were asked: 'How is your health in general' and were required to tick one of 'very good', 'good', 'fair', 'bad', or 'very bad'. Self-rated health is a known predictor of mortality.[23] The question itself is a previously validated way of estimating the general state an individual's health and has been used in population-based health surveys.[24,25] For the analysis this variable was dichotomized and 'very good' and 'good' categories were recoded and 'good health', and the 'fair', 'bad', and 'very bad' categories were recoded into 'less than good' state of physical health. Participants who selected the 'don't know' option were recoded as missing

For peer review only - http://bmjopen.bmj.com/site/about/guidelines.xhtml

values. Socio-economic status was estimated using a validated generic measure that can be applicable to heterogeneous settings. Participants were required to respond 'yes' or 'no' to the question 'do you have enough money for daily expenses, e.g. accommodation, travel, clothing, food?'. This approach was considered suitable as different UAs use dissimilar indices to express socio-economic status or deprivation in their locales which may not be comparable to those of other settings. The level of education was categorised as either 'no formal education or below', 'primary education', 'secondary education', or 'university education'.

Data analysis methods.

One-month period prevalence of LBP was estimated with associated 95% confidence intervals (95% CI) for all participating countries and cities. The prevalence was further analysed by age and sex. Chi-squared hypothesis tests were used to estimate statistical significance for all categorical comparisons. The prevalence of PD was estimated as a dichotomized variable (a score of ≥4 representing distress – a standardised cut-off). To explore the relationship between LBP and physical and mental health, unconditional logistic regression analyses were carried out. Univariable logistic regression was used to summarise the relationship between (i) LBP and self-rated health, and (ii) LBP and PD. Multivariable logistic regression was carried out adjusting for age, sex, educational level, and socio-economic status. Comparisons of these associations between settings were illustrated using high-low-close charts. All analyses were done with SPSS version 22, Microsoft Excel and Microsoft PowerPoint version 2013.

Patient and Public Involvement

Being a secondary analysis of an already existing dataset, the public and patients were not involved in the design of this study.

For peer review only - http://bmjopen.bmj.com/site/about/guidelines.xhtml

RESULTS

Distribution of Low back pain by place and person.

The one-month period prevalence of LBP in Europe was 44.6% (95% CI: 43.87 – 45.33). The prevalence ranged from 33.4% (95% CI: 30.21 – 36.59) in Norway to 67.7% (95% CI: 65.26 – 70.14) in Lithuania (figure 1.0). The prevalence in Lithuania, Slovenia, Germany, Slovakia, and Romania were all statistically significantly higher than the European average. As shown also, the prevalence of LBP in the UK, Macedonia, The Netherlands, and Norway were all significantly lower than the European prevalence. Although the prevalence in France was lower than the European prevalence, it did not achieve statistical significance.

Prevalence varied widely across the EURO URHIS 2 cities (table A supplementary material). Approximately two out of three adults in eastern European cities such as Siauliai, Kaunas, and Maribor reported LBP whereas just about one in three reported symptoms Stockport, Birmingham, and Skopje. Noticeably, the cities whose LBP prevalence fell below the European average were dominated by UAs within the United Kingdom. Of all UK cities included in the survey, only Knowsley had a prevalence rate greater than the European average.

Prevalence was statistically significantly higher for women (48.2%) than for men (41%) (table B supplementary material). The same pattern was consistent across most of the EURO URHIS 2 countries the only exception being Macedonia. Although male prevalence was higher in Macedonia, this was not statistically significant. Female prevalence in Romania, Turkey, UK, and the Netherlands were statistically significantly higher than those of their male counterparts. The male to female gap was widest in Turkey.

For peer review only - http://bmjopen.bmj.com/site/about/guidelines.xhtml

Generally, the prevalence of LBP significantly increased with age. The 18 – 39 age group had a prevalence of 34.8%, whilst the 40 – 59 age group had 46.3%. The highest prevalence of 48.2% was recorded by over 60s. This pattern was found to be fairly consistent across all the participating countries the only exception being Germany. Furthermore, the 18-39 age group across most of the countries recorded similar prevalence rates which was around ~30%. In the UK, the Netherlands and Norway, none of the age groups recorded prevalence rates of up to 40%. The highest differential in prevalence with respect to age was found in Romania where the prevalence doubles between the lowest and highest age categories.

On the whole, it can be seen from table 1.0 that people in the 40-59 age group had a 61% higher chance of LBP than people in the 18-39 age group. Similarly, the 60 and above age group had the highest unadjusted risk for LBP which was about 74% increased risk compared to the youngest group. The one-month period prevalence of LBP in the study was found to vary significantly with a generic measure of socio-economic status and individuals' level of education. Participants who reported not being able to meet up with their daily expenses had an almost two-fold increase in risk of LBP compared to those who could (Odds Ratio [OR]: 1.96, 95% CI: 1.83 – 2.11). Persons with just primary or secondary education had more than a two-fold increase in the risk of LBP compared to those who had university education. Whereas those with No formal education had a 44% increase in risk.

For peer review only - http://bmjopen.bmj.com/site/about/guidelines.xhtml

Table 1.0 Risks of LBP associated with socio-demographic variables.

	Experienced Low back pain in the last one month.			
	N	%	OR	95% CI
**Sex**				
Male	8774	41	1.0	-
Female	8801	48.2	1.34	1.26 – 1.42
**Age**				
18-39	4022	34.8	1.0	-
40-59	4808	46.3	1.61	1.48 – 1.76
≥ 60	8745	48.2	1.74	1.61 – 1.88
**SES**				
Able to meet daily expenses	12,569	40.6	1.0	-
Not able to	4064	57.3	1.96	1.83 – 2.11
**Education**				
University education	5721	37.0	1.0	-
Secondary education	9200	45.9	2.24	1.85 – 2.72
Primary education	1904	57.2	2.28	2.05 – 2.54
No formal education	468	56.8	1.45	1.35 – 1.55

Associated physical and mental health burden of LBP.

This study found almost a four-fold increased risk of having 'less than good' physical health in LBP sufferers compared to those who did not have LBP (shown in table 2.0). After accounting for the effects of age, sex, socio-economic status and level of education, only a mild effect modification was noticed. The adjusted Odds ratio for the association was 3.54 (95% CI: 3.31 – 3.80; p-value = .0001).

For peer review only - http://bmjopen.bmj.com/site/about/guidelines.xhtml

Table 2.0: Association between LBP and self-rated health and psychological distress.

	‘Less than good’ self-rated health					
		N	%	OR	95% CI	p
Low back pain	No	3,076	39.3	1.0	-	-
	Yes (unadjusted)	4,759	60.7	3.95	3.71 – 4.21	.0001
	Yes* (adjusted)			3.54	3.31 – 3.80	.0001
	Psychological Distress					
Low back Pain	No	2,267	49	1.0	-	-
	Yes (unadjusted)	2,355	51	1.64	1.50 – 1.78	.0001
	Yes* (adjusted)			1.44	1.32 – 1.58	.0001

*Adjusted for sex, age, socio-economic status and educational level

This association between LBP and poor self-rated physical health was consistent across all participating countries with OR ranging from 2.14 (95% CI: 1.77-3.28) in France to 4.30 (95% CI: 3.19-5.79) in Lithuania (shown in Figure 2.0). This association is strongest in the eastern European countries of Lithuania and Macedonia as well as in Germany.

There was also strong association between LBP and Psychological distress (PD) with OR of 1.64 (95% CI: 1.50 – 1.78), indicating a 64% increase in risk of PD associated with the occurrence of LBP. As shown in Table 2.0. above, after adjusting for the effects of age, sex, socio-economic status and level of education, the risk was slightly attenuated with a 44% elevated risk of PD amongst sufferers of LBP when compared to non-sufferers (95% CI: 1.32 – 1.58; p-value = .0001).

For peer review only - http://bmjopen.bmj.com/site/about/guidelines.xhtml

For peer review only

As can be seen in figure 3.0, the association between LBP and PD varies widely between countries. It ranges from being non-existent in Macedonia and Norway to statistically significant association in the Netherlands, UK, Romania, Germany, and Lithuania as indicated by their ORs and associated confidence intervals. In the latter countries, those suffering LBP had between 39% to 104% greater risk of PD compared to non-sufferers. For Turkey, France, Slovenia, and Slovakia, there was a slight increase in risk of PD amongst those suffering from LBP though not statistically significant.

Similarly, figure 4.0 shows that the association of LBP and PD across the various UAs varies widely from and OR of 0.78 (95% CI: 0.50 – 1.21) in Skopje to 2.56 (95% CI: 1.43 – 3.57) in Siauliai.

DISCUSSION

This research found that the one-month period prevalence of low back pain (LBP) in Europe was 44.6% widely ranging between countries from 33.4% - 67.7%. This finding aligns with findings from previous research assessing one-month period prevalence carried out among European populations.[26-28] A Greek cross-sectional study[28] found a prevalence of 37.1% whilst a cohort study carried out in the north-west of England reported a 39% prevalence.[26] Furthermore, a series of five cross sectional surveys conducted over a 20-year period in Finland with a cumulative response of 29,043 revealed an adult prevalence ranging between 46%-51%.[27] A similar one-month period prevalence was found by a recent systematic review.[29] However, these earlier research works were limited due to lack of comparability across heterogeneous settings. This study demonstrated a wide variation in LBP prevalence across

For peer review only - http://bmjopen.bmj.com/site/about/guidelines.xhtml

European urban areas (UAs). Considering that data on LBP was collected using a previously validated question with an optimal recall period, and that the same instrument was used in all participating cities, the differences are most likely to be real. LBP is usually managed at local general practitioners' clinics hence it is plausible that places with easier access to health services might have lower prevalence as the sufferers may receive early medical attention. This relatively high prevalence has implications for healthcare resource allocation as cities with higher proportion of their adult population suffering LBP should be expected to allocate more resources towards its care.

This research also found that the female sex, older people and people of lower socio-economic status as well as the less educated are at greatest risk of LBP (table 1.0). Although similar associations with lower socio-economic status, less education, and older age have been consistent from earlier studies,[30,31] opinion is divided on the sex differences. A notable review done for the global burden of disease study found greater prevalence in males across all regions of the world.[1] However, the studies that specifically assessed one-month period prevalence found higher proportions amongst females.[26-28]

Irrespective of sex, age, socio-economic status or level of education, this study found that adult Europeans suffering from LBP have a three-and-half-fold higher risk of experiencing a poor state of physical health when compared to non-sufferers. This association is stronger than findings from earlier studies amongst European populations. A Finnish cross sectional study reported an association between chronic pain and 'poor self-rated health' with OR ranging from 1.16 – 2.62 depending on the frequency of the pain.[32] On the other hand, a British cohort study found that background self-rated poor health is a predictor of chronicity amongst LBP users of primary care.[33] Although an OR of 3.6 (95% CI: 1.9 – 6.8) was found, estimates had wide confidence

For peer review only - http://bmjopen.bmj.com/site/about/guidelines.xhtml

For peer review only

intervals as the sample size was only 180. However, whether as predictor or consequence, no previous study has compared the strength of this association across different European settings. Being a cross-sectional survey, this association, though strong, consistent across all cities and countries, and highly significant, cannot be assumed to be causal. It is also noteworthy to observe that the pattern of this association was similar to the pattern of the prevalence of LBP across countries. Implying that countries with relatively higher prevalence of LBP also tended to have stronger association with poor physical health (figure 2.0).

Another key finding of this research is that adult urban Europeans with LBP stand a 44% higher risk of psychological distress (PD). An American longitudinal study similarly found that LBP at baseline was associated with a higher risk of PD at the 18th month review with an OR of 1.36 (95% CI: 1.07 – 1.72).[34] The research had a much smaller sample size of 681 and was restricted to only the US. But why is this association important? It is known that amongst sufferers of LBP, PD predicts response to therapy and chronicity both of which have significant resource implications.[10,13] PD also predicts occurrence of disability[11] and thus predicts poverty given that Europeans living with a disability are significantly poorer than those who are not.[35] This finding has potentially far reaching implications for planning public health interventions, resource allocation, and social services planning and delivery. More so in countries like the UK, and the Netherlands who despite having a comparatively low prevalence of LBP (figure 1.0), have significantly higher burdens of its associated poor mental health (figure 3.0). The same goes for urban areas such as Wirral, Craiova, and Tameside and Glossop three of which have relatively low LBP prevalence but higher burdens of PD associated LBP (figure 4.0).

Furthermore, the distribution of LBP in urban Europe varied differently from the distribution of its associated mental health burden. Implying that prevalence figures alone do not represent the

For peer review only - http://bmjopen.bmj.com/site/about/guidelines.xhtml

associated mental health burden of LBP and likewise may not represent its other associated burdens and impact. Therefore, health policy makers will benefit greatly from understanding the associated burdens of LBP in the context of particular UAs to help fashion appropriate interventions amongst high-risk and general populations.

Globally, Low back pain is more prevalent in high income countries than in low and middle income countries.[36] However, within the relatively affluent European countries, this study shows that it is the female sex, the older people, the less educated and the poorer people that mostly bear its burden. Low back pain has thus become an embodiment of engrained social mechanisms that pattern health and determinants of health in urban European societies. It has long been held that these mechanisms are not inevitable but remediable.[37] Governments at national and city levels are thus expected to make policies that will protect the vulnerable members of society from the worst effects of LBP.

Expectedly, the associated burden of LBP on physical health travels in much the same direction as its prevalence. However, its associated mental health burdens are a bit subtler. Areas of relatively low LBP prevalence may bear much higher burdens of its associated mental health effects. This again, may be a pointer to underlying intersection of factors prevalent in those urban settings. Much as such factors deserve further exploration through research, the findings thus far should inform public health policy and action as well as the (re)structuring of social services. LBP associated with psychological distress is more likely to result in disability and eventually, poverty. Added to the demands of seeking and accessing care, families run a higher risk of being driven into further impoverishment. This potentially widens the inequalities gap in societies creating a vicious circle of disease, disability and disadvantage.

For peer review only - http://bmjopen.bmj.com/site/about/guidelines.xhtml

ACKNOWLEDGEMENTS

The authors acknowledge all the members of the European Urban Health Indicators Survey 2 research team.

FUNDING STATEMENT

This work was supported by [The Commonwealth Scholarship Commission] grant number [NGSS-184-2015]

COMPETING INTERESTS

The authors have no competing interests to declare.

ETHICS

The EURO-URHIS 2 research received ethical approval from the UK Research Ethics Committee (REC), The National Information Governance Board (NIGB), and the R&D governance of the local NHS organisations in each of the UK urban areas. All the participating cities received ethical approval from relevant local authorities. A Written permission was obtained from the research team for the conduct of our analysis of the data.

CONTRIBUTORSHIP STATEMENT

CU and DP contributed to the conceptualization and the design of this study, DP facilitated the acquisition of the data, CU and DP contributed to the analysis and interpretation of the data, both authors contributed to the drafting and proofreading of the manuscripts and have approved of its submission.

DATA SHARING

For peer review only - http://bmjopen.bmj.com/site/about/guidelines.xhtml

The full dataset associated with this study is held by the EURO URHIS 2 study team and can be made available upon reasonable request by an email to: danpope@liverpool.ac.uk

For peer review only

For peer review only - http://bmjopen.bmj.com/site/about/guidelines.xhtml

REFERENCES

1 Hoy D, March L, Brooks P, et al. The global burden of low back pain: estimates from the Global Burden of Disease 2010 study. *Ann Rheum Dis* 2014 Jun;73(6):968-974.

2 Murray CJ, Vos T, Lozano R, et al. Disability-adjusted life years (DALYs) for 291 diseases and injuries in 21 regions, 1990–2010: a systematic analysis for the Global Burden of Disease Study 2010. *The lancet* 2013;380(9859):2197-2223.

3 Croft PR, Macfarlane GJ, Papageorgiou AC, et al. Outcome of low back pain in general practice: a prospective study. *Br Med J* 1998 May 2;316(7141):1356-1359.

4 Dagenais S, Caro J, Haldeman S. A systematic review of low back pain cost of illness studies in the United States and internationally. *The Spine journal* 2008;8(1):8-20.

5 Maetzel A, Li L. The economic burden of low back pain: a review of studies published between 1996 and 2001. *Best Practice & Research Clinical Rheumatology* 2002;16(1):23-30.

6 Lewandowski W, Morris R, Draucker CB, et al. Chronic pain and the family: Theory-driven treatment approaches. *Issues Ment Health Nurs* 2007;28(9):1019-1044.

7 Kovacs FM, Abraira V, Zamora J, et al. Correlation between pain, disability, and quality of life in patients with common low back pain. *Spine* (Phila Pa 1976) 2004 Jan 15;29(2):206-210.

8 Koes B, Van Tulder M, Thomas S. Diagnosis and treatment of low back pain. *Br Med J* 2006;7555:1430.

9 Van Tulder MW, Scholten RJ, Koes BW, et al. Nonsteroidal anti-inflammatory drugs for low back pain: a systematic review within the framework of the Cochrane Collaboration Back Review Group. *Spine* 2000;25(19):2501-2513.

10 Gurung T, Ellard DR, Mistry D, et al. Identifying potential moderators for response to treatment in low back pain: A systematic review. *Physiotherapy* 2015;101(3):243-251.

11 Lee H, Hubscher M, Moseley GL, et al. How does pain lead to disability? A systematic review and meta-analysis of mediation studies in people with back and neck pain. *Pain* 2015 Jun;156(6):988-997.

12 Pincus T, Vlaeyen JW, Kendall NA, et al. Cognitive-behavioral therapy and psychosocial factors in low back pain: directions for the future. *Spine* (Phila Pa 1976) 2002 Mar 1;27(5):E133-8.

13 Pincus T, Burton AK, Vogel S, et al. A systematic review of psychological factors as predictors of chronicity/disability in prospective cohorts of low back pain. *Spine* 2002;27(5):109-120.

For peer review only - http://bmjopen.bmj.com/site/about/guidelines.xhtml

14 Vargas-Prada S, Serra C, Martinez JM, et al. Psychological and culturally-influenced risk factors for the incidence and persistence of low back pain and associated disability in Spanish workers: findings from the CUPID study. *Occup Environ Med* 2013 Jan;70(1):57-62.

15 Gunzburg R. Low back pain: in search of goddess Panacea. *Eur Spine J* 2014;23(10):2027.

16 Verma A, van Ameijden E, Birt C, et al. EURO-URHIS 1 and 2 consortia. Why investigate urban health indicators? *Eur J Public Health* 2015 Jul 21.

17 Economic and Social Affairs, United Nations. World Urbanization Prospects The 2007 Revision Highlights. 2008(1):230.

18 Pope D, Puzzolo E, Birt C, et al. Collecting standardised urban health indicator data at an individual level for adults living in urban areas: methodology from EURO-URHIS 2. *Eur J Public Health* 2016 Jan 7.

19 Higgerson J, Birt C, van Ameijden E, et al. Defining the urban area for cross national comparison of health indicators: the EURO-URHIS 2 boundary study. *Eur J Public Health* 2015 Jul 23.

20 Dionne CE, Dunn KM, Croft PR, et al. A consensus approach toward the standardization of back pain definitions for use in prevalence studies. *Spine* (Phila Pa 1976) 2008 Jan 1;33(1):95-103.

21 Goldberg D, Williams P. User's Guide to the GHQ. Windsor, NFER Nelson 1988.

22 Pevalin DJ. Multiple applications of the GHQ-12 in a general population sample: an investigation of long-term retest effects. *Soc Psychiatr Epidemiol* 2000;35(11):508-512.

23 Idler EL, Benyamini Y. Self-rated health and mortality: a review of twenty-seven community studies. *J Health Soc Behav* 1997:21-37.

24 Bowling A. Just one question: If one question works, why ask several? *J Epidemiol Community Health* 2005 May;59(5):342-345.

25 Eriksson I, Unden AL, Elofsson S. Self-rated health. Comparisons between three different measures. Results from a population study. *Int J Epidemiol* 2001 Apr;30(2):326-333.

26 Croft PR, Papageorgiou AC, Ferry S, et al. Psychologic distress and low back pain: evidence from a prospective study in the general population. *Spine* 1995;20(24):2731-2737.

27 Heistaro S, Vartiainen E, Heliovaara M, et al. Trends of back pain in eastern Finland, 1972-1992, in relation to socioeconomic status and behavioral risk factors. *Am J Epidemiol* 1998 Oct 1;148(7):671-682.

For peer review only - http://bmjopen.bmj.com/site/about/guidelines.xhtml

28 Stranjalis G, Tsamandouraki K, Sakas DE, et al. Low back pain in a representative sample of Greek population: analysis according to personal and socioeconomic characteristics. *Spine* 2004;29(12):1355-1360.

29 Hoy D, Bain C, Williams G, et al. A systematic review of the global prevalence of low back pain. *Arthritis & Rheumatism* 2012;64(6):2028-2037.

30 Manek NJ, MacGregor AJ. Epidemiology of back disorders: prevalence, risk factors, and prognosis. *Curr Opin Rheumatol* 2005 Mar;17(2):134-140.

31 Hoy D, Brooks P, Blyth F, et al. The epidemiology of low back pain. *Best practice & research Clinical rheumatology* 2010;24(6):769-781.

32 Mäntyselkä PT, Turunen JH, Ahonen RS, et al. Chronic pain and poor self-rated health. *JAMA* 2003;290(18):2435-2442.

33 Thomas E, Silman AJ, Croft PR, et al. Predicting who develops chronic low back pain in primary care: a prospective study. *Br Med J* 1999 Jun 19;318(7199):1662-1667.

34 Hurwitz EL, Morgenstern H, Yu F. Cross-sectional and longitudinal associations of low-back pain and related disability with psychological distress among patients enrolled in the UCLA Low-Back Pain Study. *J Clin Epidemiol* 2003;56(5):463-471.

35 Hosking D. Staying the Course: The European Disability Strategy 2010–2020. *European yearbook of disability law* 2013;4:73-99.

36 Volinn E. The Epidemiology of Low Back Pain in the Rest of the World: A Review of Surveys in Low-and Middle-Income Countries. *Spine* 1997;22(15):1747-1754.

37 Whitehead M, Dahlgren G, World Health Organization. Levelling up (part 1): a discussion paper on concepts and principles for tackling social inequities in health. 2006.

For peer review only

Figure 1.0 Prevalence of LBP with 95% CI in the EURO URHIS 2 Countries.

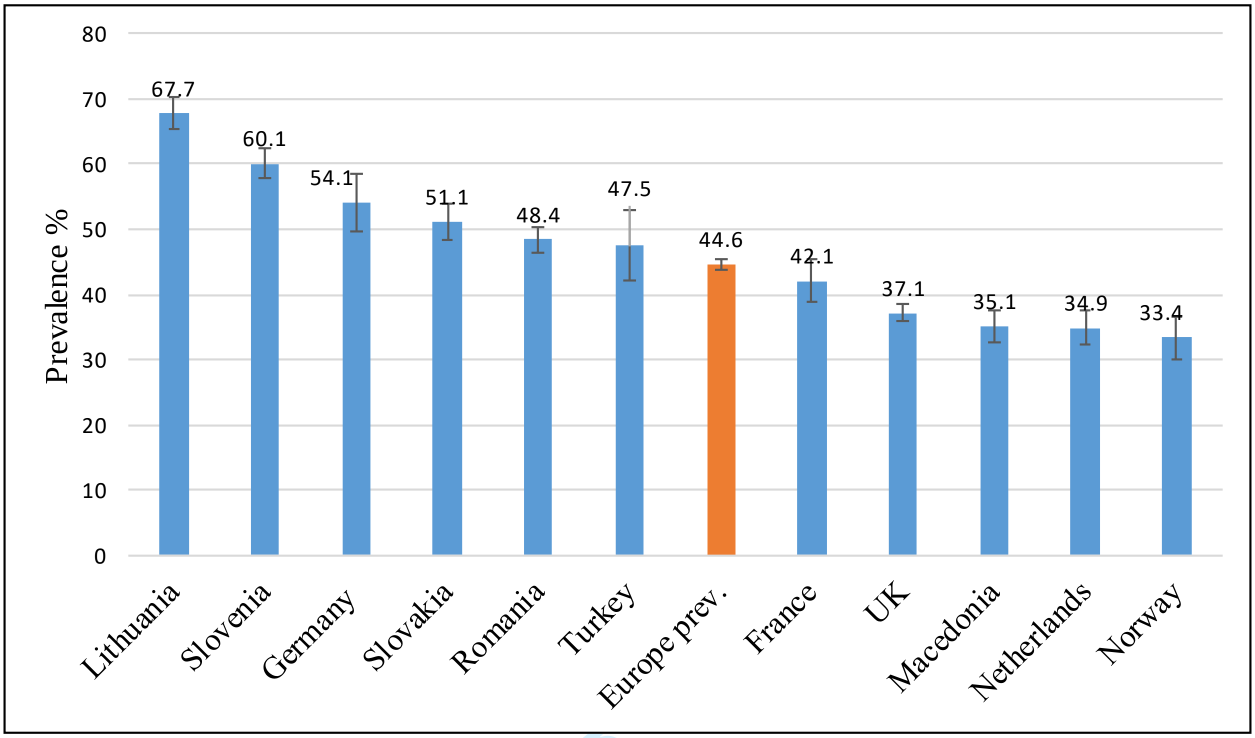

review only

Figure 2.0: Association between LBP and self-rated 'less than good' health

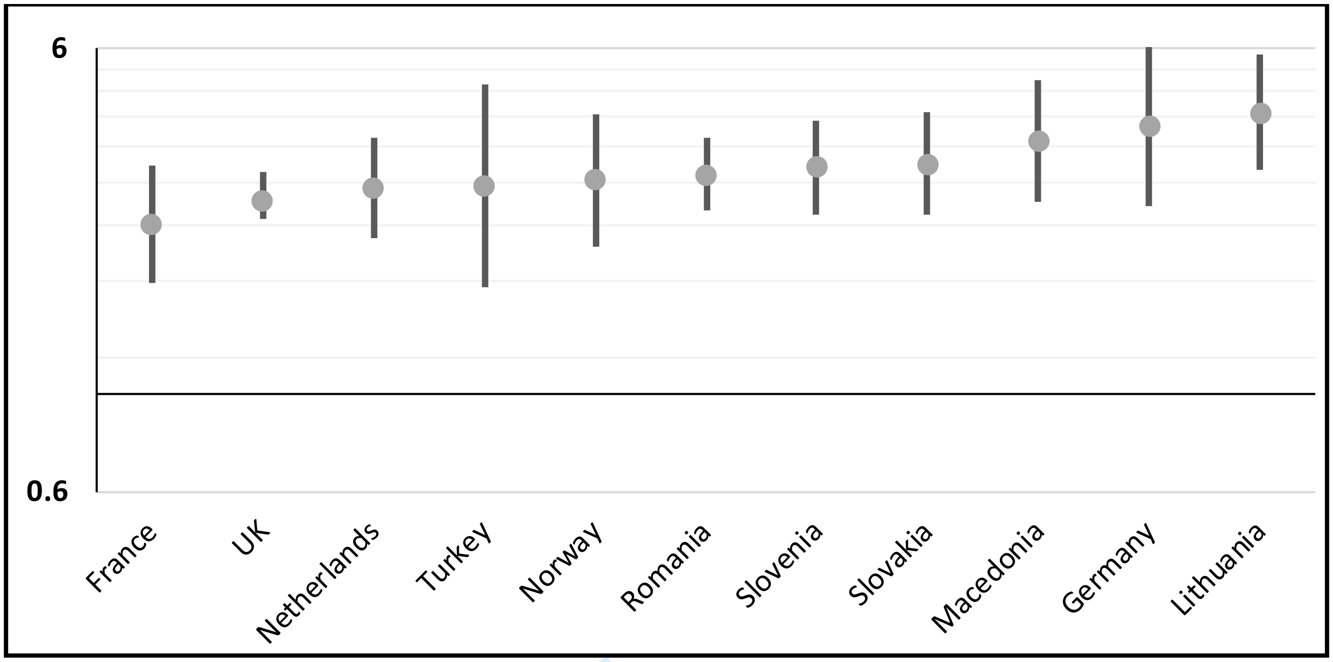

High = Upper limit of 95% Confidence Interval, **Low** = Lower limit of 95% confidence Interval

• **Close** = Adjusted Odds ratio of the Association between Low back pain and 'less than good' self-rated health

review only

Figure 3.0: Association between LBP and PD in the EURO URHIS 2 Countries

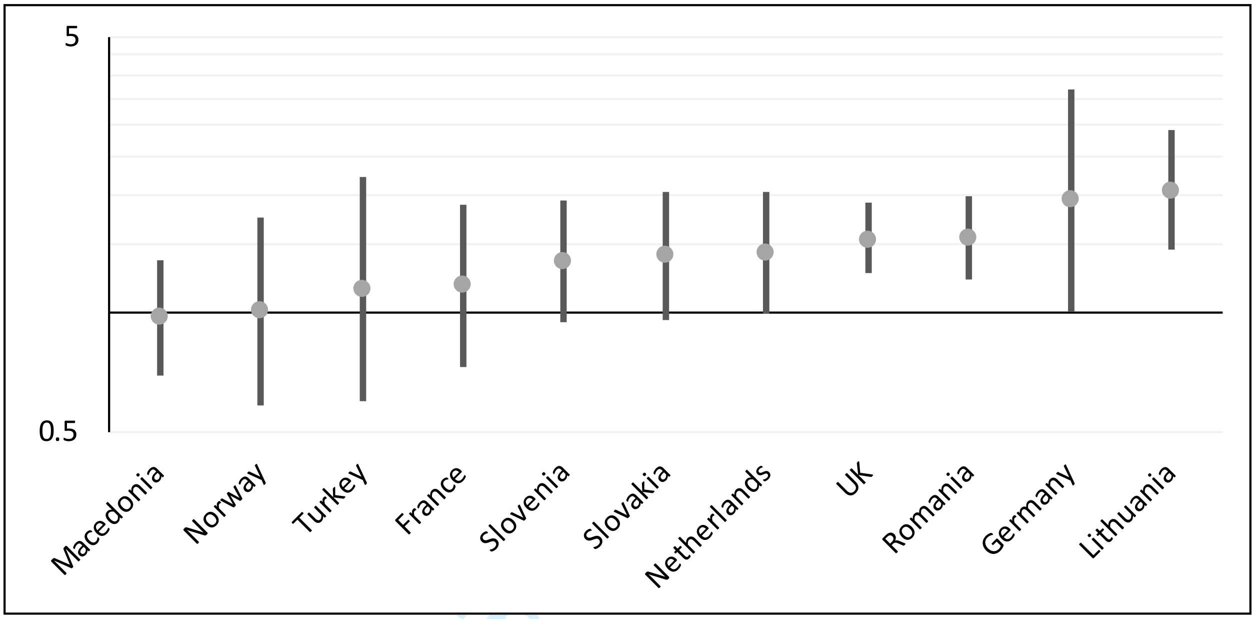

High = Upper limit of 95% Confidence interval; **Low** = Lower limit of 95% Confidence Interval

● Close value = Adjusted Odds ratio of the association between low back pain and 'Psychological distress'

Figure 4.0: Association between LBP and PD in the EURO URHIS 2 cities

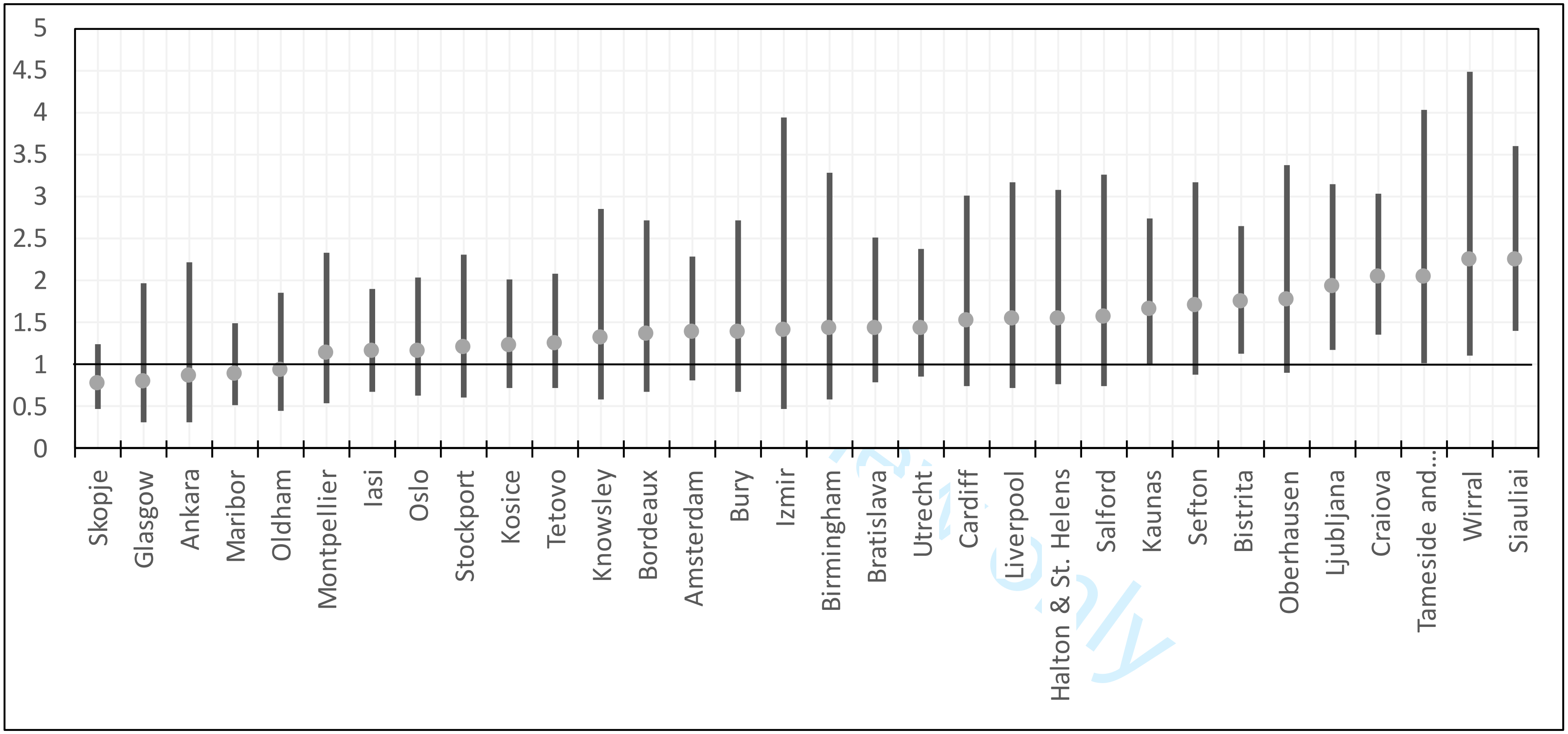

High = Upper limit of 95% Confidence Interval

Low = Lower limit of 95% confidence Interval

• **Close** = Adjusted Odds ratio of the Association between Low back pain and Psychological distress

Table A Supplementary Material

Prevalence of LBP in the EURO URHIS 2 UAs with 95% CIs

Urban Area	N	Experienced LBP in the past one month		
		%	95% CI	
			Lower CI	Upper CI
Amsterdam	663	35.7	32.05	39.35
Ankara	180	48.3	41.00	55.60
Birmingham	291	31.6	26.20	36.94
Bistrita	926	51.9	48.68	55.12
Bordeaux	443	41.1	36.52	45.68
Bratislava	547	53.9	49.72	58.08
Cardiff	437	35.7	31.21	40.19
Craiova	848	38	34.73	41.27
Glasgow	270	40.7	34.84	46.56
Bury	443	36.8	32.31	41.29
Oldham	437	38.9	34.33	43.47
Salford	399	35.6	30.90	40.30
Stockport	534	32.2	28.24	36.16
Tameside and Glossop	472	37.9	33.52	42.28
Iasi	751	55.8	52.25	59.35
Izmir	136	46.3	37.92	54.68
Kaunas	691	67.6	64.11	71.09
Kosice	689	48.8	45.07	52.53
Ljubljana	775	55.6	52.10	59.10
Maribor	813	64.3	61.01	67.59
Halton & St. Helens	384	36.5	31.68	41.32
Knowsley	412	46.6	41.78	51.42
Liverpool	440	37.5	32.98	42.02
Sefton	557	37.9	33.87	41.93
Wirral	395	34.4	29.72	39.08
Montpellier	446	43	38.41	47.59
Oberhausen	514	54.1	49.79	58.41
Oslo	809	33.4	30.15	36.65
Siauliai	685	67.7	64.20	71.20
Skopje	764	31.3	28.01	34.59
Tetovo	693	39.2	35.57	42.83
Utrecht	731	34.1	30.66	37.54

For peer review only

Table B Supplementary material

Adult Male/Female Prevalence of Low back pain in the EURO URHIS 2 Study.

Country				Low back pain	95% CI		P value
			Total	Percentage with LBP	Lower CI	Upper CI	p
UK	Sex	Male	2666	34.7	32.89	36.51	.0001
		Female	2805	39.3	37.49	41.11	
Netherlands		Male	654	30.4	26.87	33.93	.001
		Female	740	38.8	35.29	42.31	
Turkey		Male	179	39.7	32.53	46.87	.001
		Female	137	57.7	49.43	65.97	
Romania		Male	1137	41.6	38.74	44.46	.0001
		Female	1388	54.0	51.38	56.62	
France		Male	429	39.6	34.97	44.23	.154
		Female	460	44.3	39.76	48.84	
Slovakia		Male	598	47.8	43.80	51.80	.028
		Female	638	54.1	50.23	57.97	
Lithuania		Male	595	65.0	61.97	68.83	.07
		Female	781	69.7	66.48	72.92	
Slovenia		Male	785	58.3	54.85	61.75	.164
		female	803	61.8	58.44	65.16	
Germany		Male	233	50.6	44.18	57.02	.154
		female	281	56.9	51.11	62.69	
Norway		Male	414	30.2	25.78	34.62	.049
		Female	395	36.7	31.95	41.45	
Macedonia		Male	1084	35.3	32.46	38.14	.723
		Female	373	34.3	29.48	39.12	
Europe Total		Male	8774	41.0	39.97	42.03	.0001
		Female	8801	48.2	47.16	49.24	
**Total**			17575	44.6	43.87	45.33	

BMJ Open

Quantifying the association between psychological distress and low back pain in Urban Europe: A secondary analysis of a large cross-sectional study.

Journal:	*BMJ Open*
Manuscript ID	bmjopen-2020-047103.R1
Article Type:	Original research
Date Submitted by the Author:	04-May-2021
Complete List of Authors:	Ugwu, Chukwuebuka; World Health Organization Country Office for Nigeria, Communicable and Non-Communicable Diseases Cluster<br>Pope, Daniel; University of Liverpool, Department of Public Health
**Primary Subject Heading**:	Epidemiology
Secondary Subject Heading:	Public health, Mental health
Keywords:	EPIDEMIOLOGY, PUBLIC HEALTH, MENTAL HEALTH

SCHOLARONE™
Manuscripts

I, the Submitting Author has the right to grant and does grant on behalf of all authors of the Work (as defined in the below author licence), an exclusive licence and/or a non-exclusive licence for contributions from authors who are: i) UK Crown employees; ii) where BMJ has agreed a CC-BY licence shall apply, and/or iii) in accordance with the terms applicable for US Federal Government officers or employees acting as part of their official duties; on a worldwide, perpetual, irrevocable, royalty-free basis to BMJ Publishing Group Ltd ("BMJ") its licensees and where the relevant Journal is co-owned by BMJ to the co-owners of the Journal, to publish the Work in this journal and any other BMJ products and to exploit all rights, as set out in our licence.

The Submitting Author accepts and understands that any supply made under these terms is made by BMJ to the Submitting Author unless you are acting as an employee on behalf of your employer or a postgraduate student of an affiliated institution which is paying any applicable article publishing charge ("APC") for Open Access articles. Where the Submitting Author wishes to make the Work available on an Open Access basis (and intends to pay the relevant APC), the terms of reuse of such Open Access shall be governed by a Creative Commons licence – details of these licences and which Creative Commons licence will apply to this Work are set out in our licence referred to above.

Other than as permitted in any relevant BMJ Author's Self Archiving Policies, I confirm this Work has not been accepted for publication elsewhere, is not being considered for publication elsewhere and does not duplicate material already published. I confirm all authors consent to publication of this Work and authorise the granting of this licence.

For peer review only

TITLE PAGE

TITLE: Quantifying the association between psychological distress and low back pain in Urban Europe: A secondary analysis of a large cross-sectional Study.

CORRESPONDING AUTHOR:

Chukwuebuka Immanuel UGWU (MBBS, MPH)

WHO Field Office, Jerome Udoji Secretariat, Awka Anambra State, Nigeria.

Email: mazibukaz@gmail.com

CO-AUTHOR:

Daniel Pope (BSc. (hons) MSc., PhD)

Professor of Global Public Health and Epidemiology, Department of Public Health and Policy, Institute of Population Health Sciences, University of Liverpool, UK.

Email: danpope@liverpool.ac.uk

WORD COUNT: 3,422

For peer review only

For peer review only - http://bmjopen.bmj.com/site/about/guidelines.xhtml

ABSTRACT

Objectives.

This study aims to estimate the prevalence of low back pain in Europe and to quantify its associated mental and physical health burdens among adults in European urban areas.

Design.

This research is a secondary analysis of data from a large multi-country population survey.

Setting.

The population survey on which this analysis is based was conducted in 32 European urban areas across 11 countries.

Participants.

The dataset for this study was collected during the European Urban Health Indicators System 2 survey. There were a total of 19, 441 adult respondents but data from 18,028, 50.2% female (9,050) and 49.8% Male (8,978) were included in these analyses.

Primary and secondary outcome measures

Being a survey, data on the exposure (low back pain) and outcomes were collected simultaneously. The primary outcomes for this study are psychological distress and poor physical health.

Results.

The overall European Prevalence of low back pain was 44.6% (43.9-45.3) widely ranging from 33.4% in Norway to 67.7% in Lithuania. After accounting for sex, age, socio-economic status,

For peer review only

and formal education, adults in urban Europe suffering low back pain had higher odds of psychological distress aOR 1.44 (1.32-1.58) and poor self-rated health aOR 3.54 (3.31-3.80). These associations varied widely between participating countries and cities.

Conclusion.

Prevalence of low back pain, and its associations with poor physical and mental health, varies across European urban areas.

KEY WORDS

Low back pain, self-rated health, psychological distress, urban Europe,

STRENGTHS AND LIMITATIONS OF THE STUDY

- A key strength of this study is that it is based on uniformly collected data from a large population survey conducted in 32 European cities.
- Given that exposure and outcome data were collected simultaneously, our study is unable to establish a temporal sequence.
- Our analysis does not explore a dose-response relationship between low back pain and psychological distress.
- All variables included in this analysis are comparable across the different settings.

For peer review only - http://bmjopen.bmj.com/site/about/guidelines.xhtml

INTRODUCTION

In the past few decades, low back pain (LBP) has become increasingly recognised as a huge public health concern whose impact is expected to grow as life expectancy continues to improve around the world. At present, it is a leading cause of disability [1] and ranks among the top ten causes of overall disease burden globally.[2] LBP represents a substantial burden to both society and the individual. The costs to society include associated utilisation of primary care and other health services for prolonged treatments, complex physical and surgical interventions, resulting in enormous financial costs to health services.[3,4] LBP is also a major reason for temporary or permanent shortages in the workforce through sickness absence with sufferers requiring disability benefits, having a direct negative effect on the economy.[5]

In terms of burden to individuals, there is an increased need for supportive care and reduced incomes following worklessness, LBP can thus constitute a strain on family resources.[6] Also, it significantly interferes with a person's ability to engage in meaningful active social life predisposing sufferers to further morbidity and reducing individuals' overall quality of life.[7] Despite increasing treatment options such as pharmaceutical options, physical therapy, spinal manipulation and surgery,[8] improvements in outcomes may be modest at best.[9]

Psychological distress is a known mental health problem quite common in modern populations. It has been shown to play a key role in both the aetiologic and prognostic courses of LBP in adults.[10-12] Psychological distress impacts greatly on response to LBP therapy, [10] development of disability,[11] and chronicity[13] of LBP all of which have significant economic implications.[5] Conversely, background psychological distress is a recognised predictor of LBP in adults.[14]

For peer review only - http://bmjopen.bmj.com/site/about/guidelines.xhtml

Hitherto, however, there has not been any robust cross-national study exploring Psychological distress associated with LBP. Previous attempts had limited comparability across national borders due to the heterogeneity of definitions and data collection tools, differing baseline and outcome measures as well as issues with study design,[15] limiting their usefulness. This study aims to use a large, standardised, uniformly collected dataset to estimate the burden of low back pain across Europe as well as its associated mental health burden. In addition, although LBP affects all population settings, the burden is expected to differ in urban settings.[1] This is because urbanisation is associated with lifestyle changes that affect disease burden[16] and like LBP, urbanisation trend is expected to rise in the coming decades.[17] Again, no study has specifically explored Psychological distress associated LBP among urban dwellers. Our key research question is: amongst adults dwelling in urban Europe, what is the risk of psychological distress in those with low back pain compared to those without low back pain.

METHODS

Overview of EURO URHIS 2 Survey setting.

This research is based on data from the second European Urban Health Indicator System Survey (EURO-URHIS 2) conducted in 2011. The initiative was developed in an attempt to build an urban health indicator system that will help to understand the health of urban populations across Europe.[18] The project set out to develop and utilise standardised health survey tools and methodologies which would be applicable to heterogeneous Urban areas within the region. A key objective was to ensure the collection of reliably comparable data across urban areas on five key domains of urban health and determinants of health which include: demography; lifestyle; health status; health service utilisation and environmental health.[18]

For peer review only - http://bmjopen.bmj.com/site/about/guidelines.xhtml

32 urban areas across 11 countries participated in the study[18] France (Bordeaux, Montpellier), Germany (Oberhausen), Lithuania (Kaunas, Siauliai), Macedonia (Skopje, Tetovo), Norway (Oslo), Romania (Bistrita, Craiova, Iasi), United Kingdom (Birmingham, Cardiff, Glasgow, Greater Manchester – 5 urban areas, Merseyside – 5 urban areas), Slovenia (Ljubljana, Bratislava, Maribor, Kosice), Turkey (Ankara, Izmir), The Netherlands (Amsterdam, Utrecht). The EURO-URHIS 2 project clearly defined comparable boundaries applicable to participating urban areas avoiding the problem of mis-classifying sparsely populated urban areas and densely populated villages. [19] Standard population-based survey methods were used in the adults' research on which this work is based. Representative random samples stratified by age (18 – 64; ≥65) and sex (male; female) were obtained from available population registers in each urban area. Other methodological details of the EURO-URHIS 2 including the validation processes of data collection tools and the multi-lingual translations have been published elsewhere.[18]

Measurement of key variables

The exposure (predictor) variable for this study was low back pain whilst psychological distress and self-rated health were the main outcome variables. Data was also collected on potential socio-demographic confounders known to have a relationship with the predictor. These include: age, sex, level of formal education attained, and socio-economic status. All these were accounted for through multivariable regression.

The one-month period prevalence of low back pain (LBP) was assessed using a pre-validated question on back pain.[20] Individuals were asked: 'In the past month have you had low back pain which lasted one day or longer?' with the option of indicating 'yes' or 'no'. The dependent variable Psychological Distress was measured using the 12 – item General Health Questionnaire (GHQ).[21] The GHQ is a validated population-based screening tool for Psychological

For peer review only - http://bmjopen.bmj.com/site/about/guidelines.xhtml

distress.[22] It is considered to be the gold standard in assessing psychological distress and thus has been widely used in LBP[23,24] and non-LBP surveys.[22] Individuals were required to answer each of the 12 questions on the GHQ using a four-point severity scale to assess psychological distress. Before analysing of the psychological distress variable, the four possible responses were dichotomised into negative (scores 1 and 2) and positive (scores 3 and 4) according to severity. So, on each item of the GHQ, a respondent will then have either 'negative' or 'positive'. Thereafter, an overall score was computed for each person with a lowest of zero and a highest possible score of 12. Overall psychological distress score equal or greater than 4 was considered significant to yield a binary outcome with scores 0 – 3 'No Psychological distress' and scores 4 – 12 'Psychological distress'.

The state of physical health was assessed with a 'Likert-type scale' self-rated health question in which participants were asked: 'How is your health in general' and were required to tick one of 'very good', 'good', 'fair', 'bad', or 'very bad'. Self-rated health is a known predictor of mortality.[23] The question itself is a previously validated way of estimating the general state an individual's health and has been used in population-based health surveys.[24,25] For the analysis this variable was dichotomized and 'very good' and 'good' categories were recoded and 'good health', and the 'fair', 'bad', and 'very bad' categories were recoded into 'less than good' state of physical health. Participants who selected the 'don't know' option were recoded as missing values. Socio-economic status was estimated using a validated generic measure that can be applicable to heterogeneous settings. Participants were required to respond 'yes' or 'no' to the question 'do you have enough money for daily expenses, e.g., accommodation, travel, clothing, food?'. This approach was considered suitable as different UAs use dissimilar indices to express socio-economic status or deprivation in their locales which may not be comparable to those of

For peer review only - http://bmjopen.bmj.com/site/about/guidelines.xhtml

other settings. The level of education was categorised as either 'no formal education or below', 'primary education', 'secondary education', or 'university education'.

Data analysis methods.

One-month period prevalence of LBP was estimated with associated 95% confidence intervals (95% CI) for all participating countries and cities. The prevalence was further analysed by age and sex. Chi-squared hypothesis tests were used to estimate statistical significance for all categorical comparisons. The prevalence of psychological distress was estimated as a dichotomized variable (a score of ≥4 representing distress – a standardised cut-off).[26] To explore the relationship between LBP and physical and mental health, unconditional logistic regression analyses were carried out. Univariable logistic regression was used to summarise the relationship between (i) LBP and self-rated health, and (ii) LBP and Psychological distress. Multivariable logistic regression was carried out adjusting for age, sex, educational level, and socio-economic status. Comparisons of these associations between settings were illustrated using high-low-close charts. All analyses were done with SPSS version 22, Microsoft Excel and Microsoft PowerPoint version 2013.

Patient and Public Involvement

Being a secondary analysis of an already existing dataset, the public and patients were not involved in the design of this study.

For peer review only - http://bmjopen.bmj.com/site/about/guidelines.xhtml

RESULTS

Distribution of Low back pain by place and person.

The one-month period prevalence of LBP in Europe was 44.6% (95% CI: 43.87 – 45.33). The prevalence ranged from 33.4% (95% CI: 30.21 – 36.59) in Norway to 67.7% (95% CI: 65.26 – 70.14) in Lithuania (figure 1.0). The prevalence in Lithuania, Slovenia, Germany, Slovakia, and Romania were all significantly higher than the overall European prevalence. As shown also, the prevalence of LBP in the UK, Macedonia, The Netherlands, and Norway were all significantly lower than the overall prevalence. Although the prevalence in France was lower than the overall prevalence, it did not achieve statistical significance.

Prevalence varied widely across the EURO URHIS 2 cities (table A supplementary material). Approximately two out of three adults in eastern European cities such as Siauliai, Kaunas, and Maribor reported LBP whereas just about one in three reported symptoms Stockport, Birmingham, and Skopje. Noticeably, the cities whose LBP prevalence fell below the overall European prevalence were dominated by urban areas within the United Kingdom. Of all UK cities included in the survey, only Knowsley had a prevalence rate greater than the overall prevalence.

Prevalence was significantly higher for women (48.2%) than for men (41%) (table B supplementary material). The same pattern was consistent across most of the EURO URHIS 2 countries the only exception being Macedonia. Although male prevalence was higher in Macedonia, this was not significant. Female prevalence in Romania, Turkey, UK, and the Netherlands were statistically significantly higher than those of their male counterparts. The male to female gap was widest in Turkey.

For peer review only - http://bmjopen.bmj.com/site/about/guidelines.xhtml

Generally, (as shown in table c supplementary material) the prevalence of LBP significantly increased with age. The 18 – 39 age group had a prevalence of 34.8%, whilst the 40 – 59 age group had 46.3%. The highest prevalence of 48.2% was recorded by over 60s. This pattern was found to be fairly consistent across all the participating countries the only exception being Germany. Furthermore, the 18-39 age group across most of the countries recorded similar prevalence rates which was around ~30%. In the UK, the Netherlands and Norway, none of the age groups recorded prevalence rates of up to 40%. The highest differential in prevalence with respect to age was found in Romania where the prevalence doubles between the lowest and highest age categories.

On the whole, it can be seen from table 1.0 that people in the 40-59 age group had a 61% higher chance of LBP than people in the 18-39 age group. Similarly, the 60 and above age group had the highest unadjusted risk for LBP which was about 74% increased risk compared to the youngest group. The one-month period prevalence of LBP in the study was found to vary significantly with a generic measure of socio-economic status and individuals' level of education. Participants who reported not being able to meet up with their daily expenses had an almost two-fold increase in risk of LBP compared to those who could (Odds Ratio [OR]: 1.96, 95% CI: 1.83 – 2.11). Persons with just primary or secondary education had more than a two-fold increase in the risk of LBP compared to those who had university education. Whereas those with No formal education had a 44% increase in risk.

For peer review only - http://bmjopen.bmj.com/site/about/guidelines.xhtml

Table 1.0 Unadjusted Odds ratios of LBP and associated socio-demographic variables.

		Experienced Low back pain in the last one month.		
	N	%	OR	95% CI
**Sex**				
Male	8774	41	Ref	-
Female	8801	48.2	1.34	1.26 – 1.42
**Age**				
18-39	4022	34.8	Ref	-
40-59	4808	46.3	1.61	1.48 – 1.76
≥ 60	8745	48.2	1.74	1.61 – 1.88
**SES**				
Able to meet daily expenses	12,569	40.6	Ref	-
Not able to	4064	57.3	1.96	1.83 – 2.11
**Education**				
University education	5721	37.0	Ref	-
Secondary education	9200	45.9	2.24	1.85 – 2.72
Primary education	1904	57.2	2.28	2.05 – 2.54
No formal education	468	56.8	1.45	1.35 – 1.55

Associated physical and mental health burden of LBP.

This study found almost a four-fold increased risk of having ‘less than good’ physical health in LBP sufferers compared to those who did not have LBP (shown in table 2.0). After accounting for the effects of age, sex, socio-economic status and level of education, only a mild attenuation was noticed. The adjusted Odds ratio for the association was 3.54 (95% CI: 3.31 – 3.80; p-value = .0001).

For peer review only - http://bmjopen.bmj.com/site/about/guidelines.xhtml

Table 2.0: Association between LBP and self-rated health and psychological distress.

	'Less than good' self-rated health					
		N	%	OR	95% CI	p
Low back pain	No	3,076	39.3	Ref	-	-
	Yes (unadjusted)	4,759	60.7	3.95	3.71 – 4.21	.0001
	Yes* (adjusted)			3.54	3.31 – 3.80	.0001
	Psychological Distress					
Low back Pain	No	2,267	49	Ref	-	-
	Yes (unadjusted)	2,355	51	1.64	1.50 – 1.78	.0001
	Yes* (adjusted)			1.44	1.32 – 1.58	.0001

*Adjusted for sex, age, socio-economic status and educational level

This association between LBP and poor self-rated physical health was consistent across all participating countries with OR ranging from 2.14 (95% CI: 1.77-3.28) in France to 4.30 (95% CI: 3.19-5.79) in Lithuania (shown in Figure 2.0). This association is strongest in the eastern European countries of Lithuania and Macedonia as well as in Germany.

There was also strong association between LBP and Psychological distress with OR of 1.64 (95% CI: 1.50 – 1.78), indicating a 64% increase in odds of Psychological distress associated with the occurrence of LBP. As shown in Table 2.0. above, after adjusting for the effects of age, sex, socio-economic status and level of education, the risk was slightly attenuated with a 44% elevated risk of PD amongst sufferers of LBP when compared to non-sufferers (95% CI: 1.32 – 1.58; p-value = .0001).

For peer review only

For peer review only - http://bmjopen.bmj.com/site/about/guidelines.xhtml

As can be seen in figure 3.0, the association between LBP and psychological distress varies widely between countries. It ranges from being non-existent in Macedonia and Norway to statistically significant association in the Netherlands, UK, Romania, Germany, and Lithuania as indicated by their ORs and associated confidence intervals. In the latter countries, those suffering LBP had between 39% to 104% greater odds of psychological distress compared to non-sufferers. For Turkey, France, Slovenia, and Slovakia, there was a slight increase in odds of psychological distress amongst those suffering from LBP though not statistically significant.

Similarly, figure 4.0 shows that the association of LBP and psychological distress across the various urban areas varies widely from and OR of 0.78 (95% CI: 0.50 – 1.21) in Skopje to 2.56 (95% CI: 1.43 – 3.57) in Siauliai.

DISCUSSION

This research found that the one-month period prevalence of low back pain (LBP) in Europe was 44.6% widely ranging between countries from 33.4% - 67.7%. This finding aligns with findings from previous research assessing one-month period prevalence carried out among European populations.[27-29] A Greek cross-sectional study[29] found a prevalence of 37.1% whilst a cohort study carried out in the north-west of England reported a 39% prevalence.[27] Furthermore, a series of five cross sectional surveys conducted over a 20-year period in Finland with a cumulative response of 29,043 revealed an adult prevalence ranging between 46%-51%.[28] A similar one-month period prevalence was found by a recent systematic review.[30] However, these earlier research works were limited due to lack of comparability across heterogeneous settings. This study demonstrated a wide variation in LBP prevalence across

For peer review only - http://bmjopen.bmj.com/site/about/guidelines.xhtml

European urban areas. Considering that data on LBP was collected using a previously validated question with an optimal recall period, and that the same instrument was used in all participating cities, the differences are most likely to be real. LBP is usually managed at local general practitioners' clinics hence it is plausible that places with easier access to health services might have lower prevalence as the sufferers may receive early medical attention. This relatively high prevalence has implications for healthcare resource allocation as cities with higher proportion of their adult population suffering LBP should be expected to allocate more resources towards its care.

This research also found that the female sex, older people and people of lower socio-economic status as well as the less educated are at greatest risk of LBP (table 1.0). Although similar associations with lower socio-economic status, less education, and older age have been consistent from earlier studies,[31,32] opinion is divided on the sex differences. A notable review done for the global burden of disease study found greater prevalence in males across all regions of the world.[1] However, the studies that specifically assessed one-month period prevalence found higher proportions amongst females.[27-29]

Irrespective of sex, age, socio-economic status or level of education, this study found that adult Europeans suffering from LBP have three-and-half-fold higher odds of experiencing a poor state of physical health when compared to non-sufferers. This association is stronger than findings from earlier studies amongst European populations. A Finnish cross sectional study reported an association between chronic pain and 'poor self-rated health' with OR ranging from 1.16 – 2.62 depending on the frequency of the pain.[33] On the other hand, a British cohort study found that background self-rated poor health is a predictor of chronicity amongst LBP users of primary care.[34] Although an OR of 3.6 (95% CI: 1.9 – 6.8) was found, estimates had wide confidence

For peer review only - http://bmjopen.bmj.com/site/about/guidelines.xhtml

intervals as the sample size was only 180. However, whether as predictor or consequence, no previous study has compared the strength of this association across different European settings. Being a cross-sectional survey, this association, though strong, consistent across all cities and countries, and highly significant, cannot be assumed to be causal. It is also noteworthy to observe that the pattern of this association was similar to the pattern of the prevalence of LBP across countries. Implying that countries with relatively higher prevalence of LBP also tended to have stronger association with poor physical health (figure 2.0).

Another key finding of this research is that adult urban Europeans with LBP stand a 44% higher risk of psychological distress. An American longitudinal study similarly found that LBP at baseline was associated with a higher risk of psychological distress at the 18th month review with an OR of 1.36 (95% CI: 1.07 – 1.72).[35] The research had a much smaller sample size of 681 and was restricted to only the US. But why is this association important? It is known that amongst sufferers of LBP, psychological distress predicts response to therapy and chronicity both of which have significant resource implications.[10,13] It also predicts occurrence of disability[11] and thus predicts poverty given that Europeans living with a disability are significantly poorer than those who are not.[36] This finding has potentially far reaching implications for planning public health interventions, resource allocation, and social services planning and delivery. More so in countries like the UK, and the Netherlands who despite having a comparatively low prevalence of LBP (figure 1.0), have significantly higher burdens of its associated poor mental health (figure 3.0). The same goes for urban areas such as Wirral, Craiova, and Tameside and Glossop three of which have relatively low LBP prevalence but higher burdens of psychological distress associated LBP (figure 4.0).

For peer review only - http://bmjopen.bmj.com/site/about/guidelines.xhtml

Furthermore, the distribution of LBP in urban Europe varied differently from the distribution of its associated mental health burden. Implying that prevalence figures alone do not represent the associated mental health burden of LBP and likewise may not represent its other associated burdens and impact. Therefore, health policy makers will benefit greatly from understanding the associated burdens of LBP in the context of particular urban areas to help fashion appropriate interventions amongst high-risk and general populations.

Globally, Low back pain is quite prevalent in high income countries and in low-and-middle income countries.[37] However, within the relatively affluent European countries, this study shows that it is the female sex, the older people, the less educated and the poorer people that are more likely to be affected by it. Low back pain has thus become an embodiment of engrained social mechanisms that pattern health and determinants of health in urban European societies. It has long been held that these mechanisms are not inevitable but remediable.[38] Governments at national and city levels are thus expected to make policies that will protect the vulnerable members of society from the worst effects of LBP.

Expectedly, the associated burden of LBP on physical health travels in much the same direction as its prevalence. However, its associated mental health burdens are a bit subtler. Areas of relatively low LBP prevalence may have a greater associated psychological distress. The factors that shape these differences may be connected to ease of access to care, as well as availability of support to persons and families affected by low back pain. This again, may be a pointer to underlying intersection of factors prevalent in those urban settings. Much as such factors deserve further exploration through research, the findings thus far should inform public health policy and action as well as the (re)structuring of social services. LBP associated with psychological distress is more likely to result in disability and eventually, poverty. Added to the demands of seeking

For peer review only

and accessing care, families run a higher risk of being driven into further impoverishment. This potentially widens the inequalities gap in societies creating a vicious circle of disease, disability and disadvantage.

The data informing this analysis was generated from a population cross-sectional study. As a result, the temporal relationship between exposure and outcomes cannot be established by our research as both information were collected at the same time. Despite rigorous efforts to minimize bias in the EURO URHIS 2 study, such surveys may retain the possibility of information bias on account of poor recall as well as selection bias from non-response. Additionally, our research is unable to explore if a dose-response relationship exists between LBP and psychological distress. Further research is required to answer these important questions.

ACKNOWLEDGEMENTS

The authors acknowledge all the members of the European Urban Health Indicators Survey 2 research team.

FUNDING STATEMENT

This work was supported by [The Commonwealth Scholarship Commission] grant number [NGSS-184-2015]

COMPETING INTERESTS

The authors have no competing interests to declare.

ETHICS

For peer review only

This research is a secondary data analysis of an existing dataset from a population-based survey. Written permission was obtained from the research team for its use.

CONTRIBUTORSHIP STATEMENT

CIU and DP contributed to the conceptualization and the design of this study, DP facilitated the acquisition of the data, CIU and DP contributed to the analysis and interpretation of the data, both authors contributed to the drafting and proofreading of the manuscripts and have approved of its submission.

DATA SHARING

The full dataset associated with this study is held by the EURO URHIS 2 study team and can be made available upon reasonable request by an email to: danpope@liverpool.ac.uk

REFERENCES

1 Hoy D, March L, Brooks P, et al. The global burden of low back pain: estimates from the Global Burden of Disease 2010 study. *Ann Rheum Dis* 2014 Jun;73(6):968-974.

For peer review only - http://bmjopen.bmj.com/site/about/guidelines.xhtml

2 Murray CJ, Vos T, Lozano R, et al. Disability-adjusted life years (DALYs) for 291 diseases and injuries in 21 regions, 1990–2010: a systematic analysis for the Global Burden of Disease Study 2010. *The lancet* 2013;380(9859):2197-2223.

3 Croft PR, Macfarlane GJ, Papageorgiou AC, et al. Outcome of low back pain in general practice: a prospective study. *Br Med J* 1998 May 2;316(7141):1356-1359.

4 Dagenais S, Caro J, Haldeman S. A systematic review of low back pain cost of illness studies in the United States and internationally. *The Spine journal* 2008;8(1):8-20.

5 Maetzel A, Li L. The economic burden of low back pain: a review of studies published between 1996 and 2001. *Best Practice & Research Clinical Rheumatology* 2002;16(1):23-30.

6 Lewandowski W, Morris R, Draucker CB, et al. Chronic pain and the family: Theory-driven treatment approaches. *Issues Ment Health Nurs* 2007;28(9):1019-1044.

7 Kovacs FM, Abraira V, Zamora J, et al. Correlation between pain, disability, and quality of life in patients with common low back pain. *Spine* (Phila Pa 1976) 2004 Jan 15;29(2):206-210.

8 Koes B, Van Tulder M, Thomas S. Diagnosis and treatment of low back pain. *Br Med J* 2006;7555:1430.

9 Van Tulder MW, Scholten RJ, Koes BW, et al. Nonsteroidal anti-inflammatory drugs for low back pain: a systematic review within the framework of the Cochrane Collaboration Back Review Group. *Spine* 2000;25(19):2501-2513.

10 Gurung T, Ellard DR, Mistry D, et al. Identifying potential moderators for response to treatment in low back pain: A systematic review. *Physiotherapy* 2015;101(3):243-251.

11 Lee H, Hubscher M, Moseley GL, et al. How does pain lead to disability? A systematic review and meta-analysis of mediation studies in people with back and neck pain. *Pain* 2015 Jun;156(6):988-997.

12 Pincus T, Vlaeyen JW, Kendall NA, et al. Cognitive-behavioral therapy and psychosocial factors in low back pain: directions for the future. *Spine* (Phila Pa 1976) 2002 Mar 1;27(5):E133-8.

13 Pincus T, Burton AK, Vogel S, et al. A systematic review of psychological factors as predictors of chronicity/disability in prospective cohorts of low back pain. *Spine* 2002;27(5):109-120.

14 Vargas-Prada S, Serra C, Martinez JM, et al. Psychological and culturally-influenced risk factors for the incidence and persistence of low back pain and associated disability in Spanish workers: findings from the CUPID study. *Occup Environ Med* 2013 Jan;70(1):57-62.

15 Gunzburg R. Low back pain: in search of goddess Panacea. *Eur Spine J* 2014;23(10):2027.

For peer review only - http://bmjopen.bmj.com/site/about/guidelines.xhtml

16 Verma A, van Ameijden E, Birt C, et al. EURO-URHIS 1 and 2 consortia. Why investigate urban health indicators? *Eur J Public Health* 2015 Jul 21.

17 Economic and Social Affairs, United Nations. World Urbanization Prospects The 2007 Revision Highlights. 2008(1):230.

18 Pope D, Puzzolo E, Birt C, et al. Collecting standardised urban health indicator data at an individual level for adults living in urban areas: methodology from EURO-URHIS 2. *Eur J Public Health* 2016 Jan 7.

19 Higgerson J, Birt C, van Ameijden E, et al. Defining the urban area for cross national comparison of health indicators: the EURO-URHIS 2 boundary study. *Eur J Public Health* 2015 Jul 23.

20 Dionne CE, Dunn KM, Croft PR, et al. A consensus approach toward the standardization of back pain definitions for use in prevalence studies. *Spine* (Phila Pa 1976) 2008 Jan 1;33(1):95-103.

21 Goldberg D, Williams P. User's Guide to the GHQ. Windsor, NFER Nelson 1988.

22 Pevalin DJ. Multiple applications of the GHQ-12 in a general population sample: an investigation of long-term retest effects. *Soc Psychiatr Epidemiol* 2000;35(11):508-512.

23 Idler EL, Benyamini Y. Self-rated health and mortality: a review of twenty-seven community studies. *J Health Soc Behav* 1997:21-37.

24 Bowling A. Just one question: If one question works, why ask several? *J Epidemiol Community Health* 2005 May;59(5):342-345.

25 Eriksson I, Unden AL, Elofsson S. Self-rated health. Comparisons between three different measures. Results from a population study. *Int J Epidemiol* 2001 Apr;30(2):326-333.

26 Lundin A, Åhs J, Åsbring N, Kosidou K, Dal H, Tinghög P, Saboonchi F, Dalman C. Discriminant validity of the 12-item version of the general health questionnaire in a Swedish case-control study. Nordic journal of psychiatry. 2017 Apr 3;71(3):171-9.

27 Croft PR, Papageorgiou AC, Ferry S, et al. Psychologic distress and low back pain: evidence from a prospective study in the general population. *Spine* 1995;20(24):2731-2737.

28 Heistaro S, Vartiainen E, Heliovaara M, et al. Trends of back pain in eastern Finland, 1972-1992, in relation to socioeconomic status and behavioral risk factors. *Am J Epidemiol* 1998 Oct 1;148(7):671-682.

29 Stranjalis G, Tsamandouraki K, Sakas DE, et al. Low back pain in a representative sample of Greek population: analysis according to personal and socioeconomic characteristics. *Spine* 2004;29(12):1355-1360.

For peer review only - http://bmjopen.bmj.com/site/about/guidelines.xhtml

30 Hoy D, Bain C, Williams G, et al. A systematic review of the global prevalence of low back pain. *Arthritis & Rheumatism* 2012;64(6):2028-2037.

31 Manek NJ, MacGregor AJ. Epidemiology of back disorders: prevalence, risk factors, and prognosis. *Curr Opin Rheumatol* 2005 Mar;17(2):134-140.

32 Hoy D, Brooks P, Blyth F, et al. The epidemiology of low back pain. *Best practice & research Clinical rheumatology* 2010;24(6):769-781.

33 Mäntyselkä PT, Turunen JH, Ahonen RS, et al. Chronic pain and poor self-rated health. *JAMA* 2003;290(18):2435-2442.

34 Thomas E, Silman AJ, Croft PR, et al. Predicting who develops chronic low back pain in primary care: a prospective study. *Br Med J* 1999 Jun 19;318(7199):1662-1667.

35 Hurwitz EL, Morgenstern H, Yu F. Cross-sectional and longitudinal associations of low-back pain and related disability with psychological distress among patients enrolled in the UCLA Low-Back Pain Study. *J Clin Epidemiol* 2003;56(5):463-471.

36 Hosking D. Staying the Course: The European Disability Strategy 2010–2020. *European yearbook of disability law* 2013;4:73-99.

37 Morris LD, Daniels KJ, Ganguli B, Louw QA. An update on the prevalence of low back pain in Africa: a systematic review and meta-analyses. BMC Musculoskeletal Disorders. 2018 Dec;19(1):1-5.

38 Whitehead M, Dahlgren G, World Health Organization. Levelling up (part 1): a discussion paper on concepts and principles for tackling social inequities in health. 2006.

For peer review only - http://bmjopen.bmj.com/site/about/guidelines.xhtml

Figure 1.0

Figure 1.0 Prevalence of LBP with 95% CI in the EURO URHIS 2 Countries.

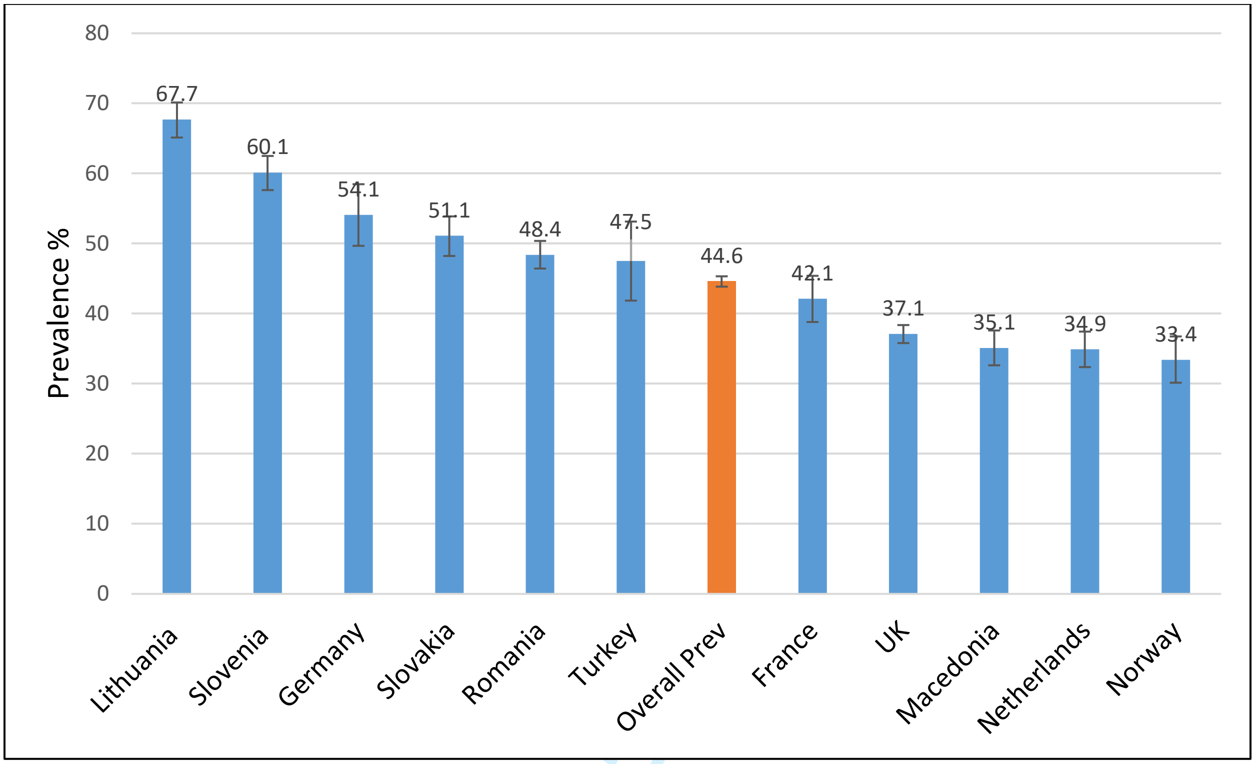

Figure 2.0

Figure 2.0: Association between LBP and self-rated 'less than good' health

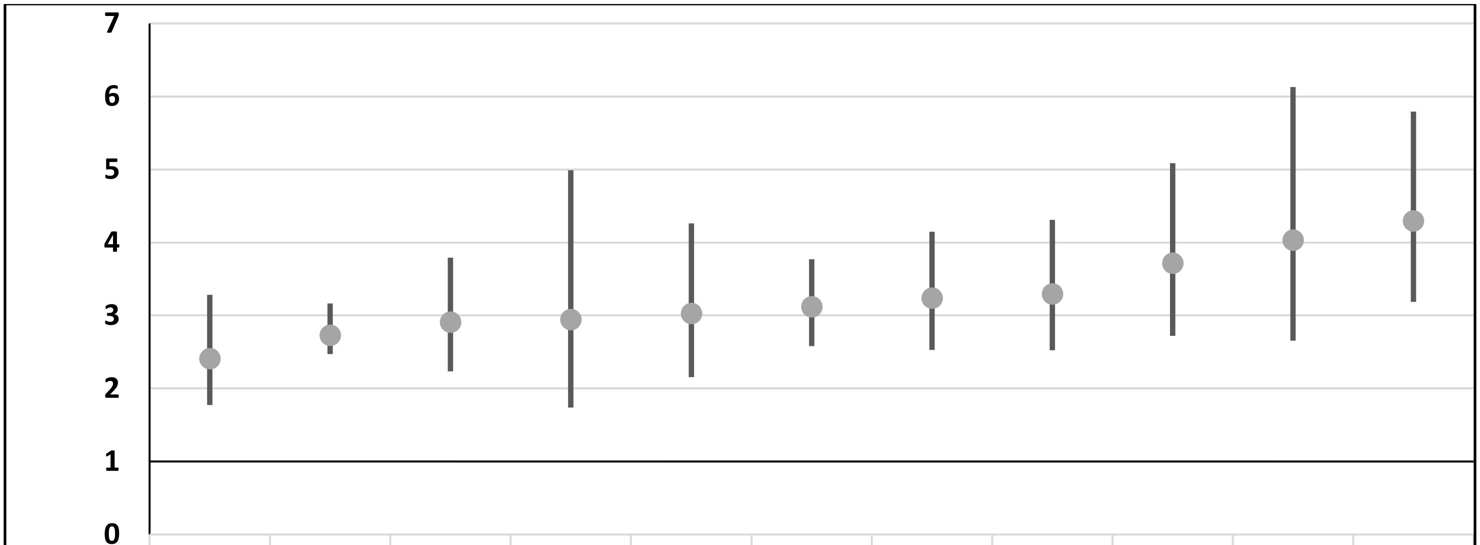

	France	UK	Netherla nds	Turkey	Norway	Romania	Slovenia	Slovakia	Macedo nia	German y	Lithuani a
High	3.281	3.166	3.793	4.988	4.261	3.769	4.148	4.309	5.084	6.129	5.794
Low	1.773	2.47	2.236	1.739	2.154	2.581	2.53	2.524	2.721	2.655	3.188
● Close	2.412	2.729	2.912	2.945	3.03	3.119	3.239	3.298	3.719	4.034	4.298

High = Upper 95% Confidence Interval, Low = Lower 95% confidence Interval

• Close = Adjusted Odds ratio of the Association between Low back pain and Psychological distress (adjusted for sex, age socio-economic status, and educational level)

Figure 3.0

Figure 3.0: Association between LBP and Psychological Distress in the EURO URHIS 2 Countries

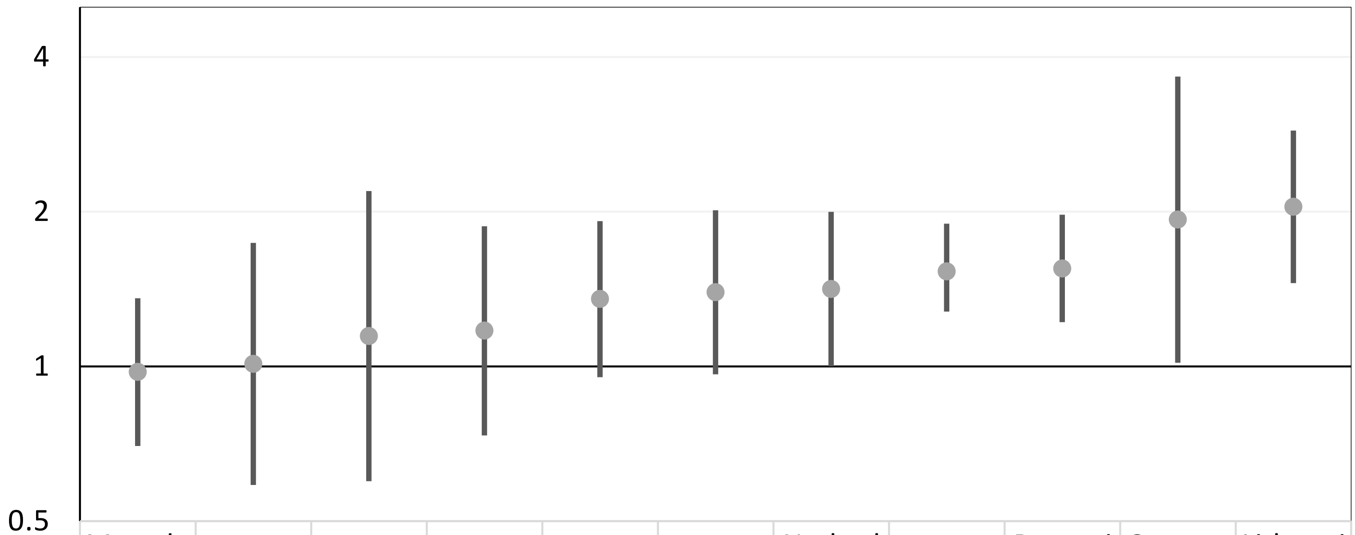

	Macedonia	Norway	Turkey	France	Slovenia	Slovakia	Netherlands	UK	Romania	Germany	Lithuania
High	1.342	1.718	2.167	1.851	1.895	1.99	1.976	1.872	1.949	3.621	2.841
Low	0.709	0.595	0.605	0.743	0.965	0.977	1.012	1.293	1.234	1.029	1.471
● Close	0.975	1.011	1.145	1.173	1.352	1.394	1.414	1.53	1.551	1.93	2.044

High value = Upper 95% Confidence interval; Low value = Lower 95% Confidence Interval

• Close value = Adjusted Odds ratio of the association between low back pain and Psychological distress (adjusted for sex, age socio-economic status, and educational level)

eview only

Figure 4.0

Figure 4.0: Association between LBP and Psychological Distress in the EURO URHIS 2 cities

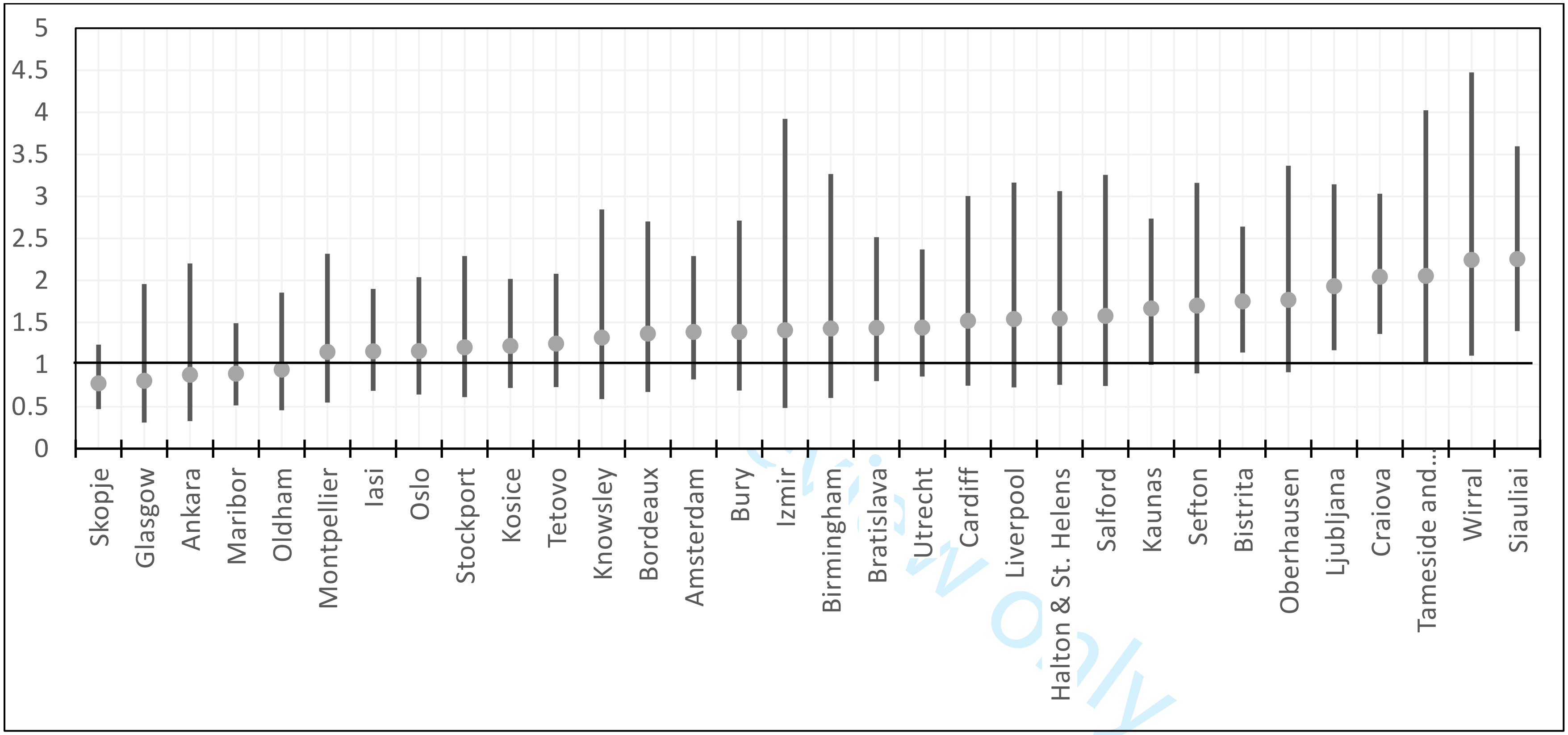

High = Upper 95% Confidence Interval

Low = Lower 95% confidence Interval

• Close = Adjusted Odds ratio of the Association between Low back pain and Psychological distress (adjusted for sex, age socio-economic status, and educational level)

Table A Supplementary Material

Prevalence of LBP in the EURO URHIS 2 UAs with 95% CIs

		Experienced LBP in the past one month			
Urban Area	N	%	95% CI		
			Lower CI	Upper CI	
Skopje	764	31.3	28.01	34.59	Macedonia
Birmingham	291	31.6	26.2	36.94	United Kingdom
Stockport	534	32.2	28.24	36.16	United Kingdom
Oslo	809	33.4	30.15	36.65	Norway
Utrecht	731	34.1	30.66	37.54	The Netherlands
Wirral	395	34.4	29.72	39.08	United Kingdom
Salford	399	35.6	30.9	40.3	United Kingdom
Amsterdam	663	35.7	32.05	39.35	The Netherlands
Cardiff	437	35.7	31.21	40.19	United Kingdom
Halton & St. Helens	384	36.5	31.68	41.32	United Kingdom
Bury	443	36.8	32.31	41.29	United Kingdom
Liverpool	440	37.5	32.98	42.02	United Kingdom
Tameside and Glossop	472	37.9	33.52	42.28	United Kingdom
Sefton	557	37.9	33.87	41.93	United Kingdom
Craiova	848	38	34.73	41.27	Romania
Oldham	437	38.9	34.33	43.47	United Kingdom
Tetovo	693	39.2	35.57	42.83	Macedonia
Glasgow	270	40.7	34.84	46.56	United Kingdom
Bordeaux	443	41.1	36.52	45.68	France
Montpellier	446	43	38.41	47.59	France
Izmir	136	46.3	37.92	54.68	Turkey
Knowsley	412	46.6	41.78	51.42	United Kingdom
Ankara	180	48.3	41	55.6	Turkey
Kosice	689	48.8	45.07	52.53	Slovakia
Bistrita	926	51.9	48.68	55.12	Romania
Bratislava	547	53.9	49.72	58.08	Slovakia
Oberhausen	514	54.1	49.79	58.41	Germany
Ljubljana	775	55.6	52.1	59.1	Slovenia
Iasi	751	55.8	52.25	59.35	Romania
Maribor	813	64.3	61.01	67.59	Slovenia
Kaunas	691	67.6	64.11	71.09	Lithuania
Siauliai	685	67.7	64.2	71.2	Lithuania

For peer review only

Table B Supplementary Material

Adult Male/Female Prevalence of Low back pain in the EURO URHIS 2 Study.

Country			Total	Low back pain<br>Percentage with LBP	95% CI<br>Lower CI	Upper CI	P value<br>p
UK	Sex	Male	2666	34.7	32.89	36.51	.0001
		Female	2805	39.3	37.49	41.11	
Netherlands		Male	654	30.4	26.87	33.93	.001
		Female	740	38.8	35.29	42.31	
Turkey		Male	179	39.7	32.53	46.87	.001
		Female	137	57.7	49.43	65.97	
Romania		Male	1137	41.6	38.74	44.46	.0001
		Female	1388	54.0	51.38	56.62	
France		Male	429	39.6	34.97	44.23	.154
		Female	460	44.3	39.76	48.84	
Slovakia		Male	598	47.8	43.80	51.80	.028
		Female	638	54.1	50.23	57.97	
Lithuania		Male	595	65.0	61.97	68.83	.07
		Female	781	69.7	66.48	72.92	
Slovenia		Male	785	58.3	54.85	61.75	.164
		female	803	61.8	58.44	65.16	
Germany		Male	233	50.6	44.18	57.02	.154
		female	281	56.9	51.11	62.69	
Norway		Male	414	30.2	25.78	34.62	.049
		Female	395	36.7	31.95	41.45	
Macedonia		Male	1084	35.3	32.46	38.14	.723
		Female	373	34.3	29.48	39.12	
Europe Total		Male	8774	41.0	39.97	42.03	.0001
		Female	8801	48.2	47.16	49.24	
**Total**			17575	44.6	43.87	45.33	

Table C Supplementary Material

Adult age prevalence of LBP in the EURO URHIS 2 Study

			Experienced LBP in the past month			
			%	95% CI		P
	Age	N		Lower CI	Upper CI	
Lithuania	18-39	301	56.5	50.90	62.10	.0001
	40-59	396	68.4	63.82	72.98	
	60+	679	72.2	68.83	75.57	
Slovenia	18-39	298	46.6	40.94	52.26	.0001
	40-59	429	60.6	55.98	65.22	
	60+	861	64.5	61.30	67.77	
Germany	18-39	79	41.8	30.92	52.68	.039
	40-59	138	59.4	51.21	67.59	
	60+	297	54.9	49.24	60.56	
Slovakia	18-39	198	33.3	26.74	39.86	.0001
	40-59	313	48.2	42.66	53.74	
	60+	725	57.1	53.50	60.70	
Romania	18-39	877	32.3	29.21	35.39	.0001
	40-59	953	51.5	48.33	54.67	
	60+	695	64.5	60.94	68.06	
Turkey	18-39	88	47.7	37.26	58.14	.936
	40-59	140	46.4	38.14	54.66	
	60+	88	48.9	38.46	59.34	
France	18-39	114	33.3	24.65	41.95	.022
	40-59	294	47.6	41.89	53.31	
	60+	481	40.7	36.31	45.09	
UK	18-39	855	31.0	27.90	34.10	.0001
	40-59	1372	37.9	35.33	40.47	
	60+	3244	38.3	36.63	39.97	
Macedonia	18-39	680	30.0	26.56	33.44	.0001
	40-59	351	28.5	23.78	33.22	
	60+	426	48.6	43.85	53.35	
Netherlands	18-39	341	29.9	25.04	34.76	.081
	40-59	268	35.4	29.67	41.13	
	60+	785	36.8	33.43	40.17	
Norway	18-39	191	30.4	23.88	36.92	.452
	40-59	154	31.8	24.44	39.16	
	60+	464	35.1	30.76	39.44	

For peer review only

STROBE Statement—Checklist of items that should be included in reports of ***cross-sectional studies***

	Item No	Recommendation
**Title and abstract**	1	(*a*) Indicate the study's design with a commonly used term in the title or the abstract Page 1 lines 2 - 3
		(*b*) Provide in the abstract an informative and balanced summary of what was done and what was found. Page 2 lines 2 – 20, page 3 lines 1 - 6
**Introduction**		
Background/rationale	2	Explain the scientific background and rationale for the investigation being reported Page 4 lines 2 – 22, page 5 lines 1 - 12
Objectives	3	State specific objectives, including any prespecified hypotheses page 5 lines 10-12
**Methods**		
Study design	4	Present key elements of study design early in the paper page 5 line 14 – 22, page 6 lines 1 - 12
Setting	5	Describe the setting, locations, and relevant dates, including periods of recruitment, exposure, follow-up, and data collection page 5 lines 14 – 22, page 6 lines 1 - 12
Participants	6	(*a*) Give the eligibility criteria, and the sources and methods of selection of participants page 6 lines 8 - 12
Variables	7	Clearly define all outcomes, exposures, predictors, potential confounders, and effect modifiers. Give diagnostic criteria, if applicable Page 6 lines 14 - 18
Data sources/ measurement	8*	For each variable of interest, give sources of data and details of methods of assessment (measurement). Describe comparability of assessment methods if there is more than one group Page 6 lines 14 – 23, page 7 lines 1 - 23
Bias	9	Describe any efforts to address potential sources of bias Page 6 lines 10 - 13
Study size	10	Explain how the study size was arrived at Not Applicable as this is a secondary analysis
Quantitative variables	11	Explain how quantitative variables were handled in the analyses. If applicable, describe which groupings were chosen and why Page 6 lines 14 – 23, page 7 lines 1 - 23
Statistical methods	12	(*a*) Describe all statistical methods, including those used to control for confounding
		(*b*) Describe any methods used to examine subgroups and interactions Page 8 lines 4 - 14
		(*c*) Explain how missing data were addressed Not Applicable
		(*d*) If applicable, describe analytical methods taking account of sampling strategy Not Applicable
		(*e*) Describe any sensitivity analyses Not Applicable
**Results**		
Participants	13*	(a) Report numbers of individuals at each stage of study—eg numbers potentially eligible, examined for eligibility, confirmed eligible, included in the study, completing follow-up, and analysed Not Applicable
		(b) Give reasons for non-participation at each stage Not Applicable
		(c) Consider use of a flow diagram Not Applicable
Descriptive data	14*	(a) Give characteristics of study participants (eg demographic, clinical, social) and information on exposures and potential confounders page 9 lines 2 – 22, page 10 lines 1 – 19, page 11 table 1.0
		(b) Indicate number of participants with missing data for each variable of interest Not Applicable

For peer review only - http://bmjopen.bmj.com/site/about/guidelines.xhtml

Outcome data	15*	Report numbers of outcome events or summary measures Figure 1.0
Main results	16	(*a*) Give unadjusted estimates and, if applicable, confounder-adjusted estimates and their precision (eg, 95% confidence interval). Make clear which confounders were adjusted for and why they were included Page 12 table 2.0
		(*b*) Report category boundaries when continuous variables were categorized Page 11 table 1.0, page 12 table 2.0
		(*c*) If relevant, consider translating estimates of relative risk into absolute risk for a meaningful time period Not Applicable
Other analyses	17	Report other analyses done—eg analyses of subgroups and interactions, and sensitivity analyses Figures 2.0, 3.0, and 4.0
**Discussion**		
Key results	18	Summarise key results with reference to study objectives Page 13 lines 13 – 22, page 14 lines 1 – 23, and page 15 lines 1 - 22
Limitations	19	Discuss limitations of the study, taking into account sources of potential bias or imprecision. Discuss both direction and magnitude of any potential bias page 17 lines 4 - 10
Interpretation	20	Give a cautious overall interpretation of results considering objectives, limitations, multiplicity of analyses, results from similar studies, and other relevant evidence Page 16 lines 16 - 17
Generalisability	21	Discuss the generalisability (external validity) of the study results Page 16 lines 1 - 17
**Other information**		
Funding	22	Give the source of funding and the role of the funders for the present study and, if applicable, for the original study on which the present article is based page 17 lines 12 - 17

*Give information separately for exposed and unexposed groups.

Note: An Explanation and Elaboration article discusses each checklist item and gives methodological background and published examples of transparent reporting. The STROBE checklist is best used in conjunction with this article (freely available on the Web sites of PLoS Medicine at http://www.plosmedicine.org/, Annals of Internal Medicine at http://www.annals.org/, and Epidemiology at http://www.epidem.com/). Information on the STROBE Initiative is available at www.strobe-statement.org.

For peer review only

BMJ Open

Quantifying the association between psychological distress and low back pain in Urban Europe: A secondary analysis of a large cross-sectional study.

Journal:	*BMJ Open*
Manuscript ID	bmjopen-2020-047103.R2
Article Type:	Original research
Date Submitted by the Author:	02-Sep-2021
Complete List of Authors:	Ugwu, Chukwuebuka; World Health Organization Country Office for Nigeria, Communicable and Non-Communicable Diseases Cluster<br>Pope, Daniel; University of Liverpool, Department of Public Health
**Primary Subject Heading**:	Epidemiology
Secondary Subject Heading:	Public health, Mental health
Keywords:	EPIDEMIOLOGY, PUBLIC HEALTH, MENTAL HEALTH

SCHOLARONE™
Manuscripts

I, the Submitting Author has the right to grant and does grant on behalf of all authors of the Work (as defined in the below author licence), an exclusive licence and/or a non-exclusive licence for contributions from authors who are: i) UK Crown employees; ii) where BMJ has agreed a CC-BY licence shall apply, and/or iii) in accordance with the terms applicable for US Federal Government officers or employees acting as part of their official duties; on a worldwide, perpetual, irrevocable, royalty-free basis to BMJ Publishing Group Ltd ("BMJ") its licensees and where the relevant Journal is co-owned by BMJ to the co-owners of the Journal, to publish the Work in this journal and any other BMJ products and to exploit all rights, as set out in our licence.

The Submitting Author accepts and understands that any supply made under these terms is made by BMJ to the Submitting Author unless you are acting as an employee on behalf of your employer or a postgraduate student of an affiliated institution which is paying any applicable article publishing charge ("APC") for Open Access articles. Where the Submitting Author wishes to make the Work available on an Open Access basis (and intends to pay the relevant APC), the terms of reuse of such Open Access shall be governed by a Creative Commons licence – details of these licences and which Creative Commons licence will apply to this Work are set out in our licence referred to above.

Other than as permitted in any relevant BMJ Author's Self Archiving Policies, I confirm this Work has not been accepted for publication elsewhere, is not being considered for publication elsewhere and does not duplicate material already published. I confirm all authors consent to publication of this Work and authorise the granting of this licence.

For peer review only

TITLE PAGE

TITLE: Quantifying the association between psychological distress and low back pain in Urban Europe: A secondary analysis of a large cross-sectional Study.

CORRESPONDING AUTHOR:

Chukwuebuka Immanuel UGWU (MBBS, MPH)

WHO Field Office, Jerome Udoji Secretariat, Awka Anambra State, Nigeria.

Email: mazibukaz@gmail.com

CO-AUTHOR:

Daniel Pope (BSc. (hons) MSc., PhD)

Professor of Global Public Health and Epidemiology, Department of Public Health and Policy, Institute of Population Health Sciences, University of Liverpool, UK.

Email: danpope@liverpool.ac.uk

WORD COUNT: 3,644

For peer review only

ABSTRACT

Objectives.

This study aims to estimate the prevalence of low back pain in Europe and to quantify its associated mental and physical health burdens among adults in European urban areas.

Design.

This research is a secondary analysis of data from a large multi-country population survey.

Setting.

The population survey on which this analysis is based was conducted in 32 European urban areas across 11 countries.

Participants.

The dataset for this study was collected during the European Urban Health Indicators System 2 survey. There were a total of 19, 441 adult respondents but data from 18,028, 50.2% female (9,050) and 49.8% Male (8,978) were included in these analyses.

Primary and secondary outcome measures

Being a survey, data on the exposure (low back pain) and outcomes were collected simultaneously. The primary outcomes for this study are psychological distress and poor physical health.

Results.

The overall European Prevalence of low back pain was 44.6% (43.9-45.3) widely ranging from 33.4% in Norway to 67.7% in Lithuania. After accounting for sex, age, socio-economic status,

For peer review only

and formal education, adults in urban Europe suffering low back pain had higher odds of psychological distress aOR 1.44 (1.32-1.58) and poor self-rated health aOR 3.54 (3.31-3.80). These associations varied widely between participating countries and cities.

Conclusion.

Prevalence of low back pain, and its associations with poor physical and mental health, varies across European urban areas.

KEY WORDS

Low back pain, self-rated health, psychological distress, urban Europe,

STRENGTHS AND LIMITATIONS OF THE STUDY

- A key strength of this study is that it is based on uniformly collected data from a large population survey conducted in 32 European cities.
- Given that exposure and outcome data were collected simultaneously, our study is unable to establish a temporal sequence.
- Our analysis does not explore a dose-response relationship between low back pain and psychological distress.
- All variables included in this analysis are comparable across the different settings.

For peer review only

For peer review only - http://bmjopen.bmj.com/site/about/guidelines.xhtml

INTRODUCTION

In the past few decades, low back pain (LBP) has become increasingly recognised as a huge public health concern whose impact is expected to grow as life expectancy continues to improve around the world. At present, it is a leading cause of disability [1] and ranks among the top ten causes of overall disease burden globally.[2] LBP represents a substantial burden to both society and the individual. The costs to society include associated utilisation of primary care and other health services for prolonged treatments, complex physical and surgical interventions, resulting in enormous financial costs to health services.[3,4] LBP is also a major reason for temporary or permanent shortages in the workforce through sickness absence with sufferers requiring disability benefits, having a direct negative effect on the economy.[5]

In terms of burden to individuals, there is an increased need for supportive care and reduced incomes following worklessness, LBP can thus constitute a strain on family resources.[6] Also, it significantly interferes with a person's ability to engage in meaningful active social life predisposing sufferers to further morbidity and reducing individuals' overall quality of life.[7] Despite increasing treatment options such as pharmaceutical options, physical therapy, spinal manipulation and surgery,[8] improvements in outcomes may be modest at best.[9]

Psychological distress is a known mental health problem quite common in modern populations. It has been shown to play a key role in both the aetiologic and prognostic courses of LBP in adults.[10-12] Psychological distress impacts greatly on response to LBP therapy, [10] development of disability,[11] and chronicity[13] of LBP all of which have significant economic implications.[5] Conversely, background psychological distress is a recognised predictor of LBP in adults.[14]

For peer review only

Hitherto, however, there has not been any robust cross-national study exploring Psychological distress associated with LBP. Previous attempts had limited comparability across national borders due to the heterogeneity of definitions and data collection tools, differing baseline and outcome measures as well as issues with study design,[15] limiting their usefulness. This study aims to use a large, standardised, uniformly collected dataset to estimate the burden of low back pain across Europe as well as its associated mental health burden. In addition, although LBP affects all population settings, the burden is expected to differ in urban settings.[1] This is because urbanisation is associated with lifestyle changes that affect disease burden[16] and like LBP, urbanisation trend is expected to rise in the coming decades.[17] Again, no study has specifically explored Psychological distress associated LBP among urban dwellers. Our key research question is: amongst adults dwelling in urban Europe, what is the risk of psychological distress in those with low back pain compared to those without low back pain.

METHODS

Overview of EURO URHIS 2 Survey setting.

This research is based on data from the second European Urban Health Indicator System Survey (EURO-URHIS 2) conducted in 2011. The initiative was developed in an attempt to build an urban health indicator system that will help to understand the health of urban populations across Europe.[18] The project set out to develop and utilise standardised health survey tools and methodologies which would be applicable to heterogeneous Urban areas within the region. A key objective was to ensure the collection of reliably comparable data across urban areas on five key domains of urban health and determinants of health which include: demography; lifestyle; health status; health service utilisation and environmental health.[18]

For peer review only - http://bmjopen.bmj.com/site/about/guidelines.xhtml

32 urban areas across 11 countries participated in the study[18] France (Bordeaux, Montpellier), Germany (Oberhausen), Lithuania (Kaunas, Siauliai), Macedonia (Skopje, Tetovo), Norway (Oslo), Romania (Bistrita, Craiova, Iasi), United Kingdom (Birmingham, Cardiff, Glasgow, Greater Manchester – 5 urban areas, Merseyside – 5 urban areas), Slovenia (Ljubljana, Bratislava, Maribor, Kosice), Turkey (Ankara, Izmir), The Netherlands (Amsterdam, Utrecht). The EURO-URHIS 2 project clearly defined comparable boundaries applicable to participating urban areas avoiding the problem of mis-classifying sparsely populated urban areas and densely populated villages. [19] Standard population-based survey methods were used in the adults' research on which this work is based. Representative random samples stratified by age (18 – 64; ≥65) and sex (male; female) were obtained from available population registers in each urban area. Other methodological details of the EURO-URHIS 2 including the validation processes of data collection tools and the multi-lingual translations have been published elsewhere.[18]

Measurement of key variables

The exposure (predictor) variable for this study was low back pain whilst psychological distress and self-rated health were the main outcome variables. Data was also collected on potential socio-demographic confounders known to have a relationship with the predictor. These include: age, sex, level of formal education attained, and socio-economic status. All these were accounted for through multivariable regression.

The one-month period prevalence of low back pain (LBP) was assessed using a pre-validated question on back pain.[20] Individuals were asked: 'In the past month have you had low back pain which lasted one day or longer?' with the option of indicating 'yes' or 'no'. The dependent variable Psychological Distress was measured using the 12 – item General Health Questionnaire (GHQ).[21] The GHQ is a validated population-based screening tool for Psychological

For peer review only - http://bmjopen.bmj.com/site/about/guidelines.xhtml

distress.[22] It is considered to be the gold standard in assessing psychological distress and thus has been widely used in LBP[23,24] and non-LBP surveys.[22] Individuals were required to answer each of the 12 questions on the GHQ using a four-point severity scale to assess psychological distress. Before analysing of the psychological distress variable, the four possible responses were dichotomised into negative (scores 1 and 2) and positive (scores 3 and 4) according to severity. So, on each item of the GHQ, a respondent will then have either 'negative' or 'positive'. Thereafter, an overall score was computed for each person with a lowest of zero and a highest possible score of 12. Overall psychological distress score equal or greater than 4 was considered significant to yield a binary outcome with scores 0 – 3 'No Psychological distress' and scores 4 – 12 'Psychological distress'.

The state of physical health was assessed with a 'Likert-type scale' self-rated health question in which participants were asked: 'How is your health in general' and were required to tick one of 'very good', 'good', 'fair', 'bad', or 'very bad'. Self-rated health is a known predictor of mortality.[23] The question itself is a previously validated way of estimating the general state an individual's health and has been used in population-based health surveys.[24,25] For the analysis this variable was dichotomized and 'very good' and 'good' categories were recoded and 'good health', and the 'fair', 'bad', and 'very bad' categories were recoded into 'less than good' state of physical health. Participants who selected the 'don't know' option were recoded as missing values. Socio-economic status was estimated using a validated generic measure that can be applicable to heterogeneous settings. Participants were required to respond 'yes' or 'no' to the question 'do you have enough money for daily expenses, e.g., accommodation, travel, clothing, food?'. This approach was considered suitable as different UAs use dissimilar indices to express socio-economic status or deprivation in their locales which may not be comparable to those of

For peer review only

other settings. The level of education was categorised as either 'no formal education or below', 'primary education', 'secondary education', or 'university education'.

Data analysis methods.

One-month period prevalence of LBP was estimated with associated 95% confidence intervals (95% CI) for all participating countries and cities. The prevalence was further analysed by age and sex. Chi-squared hypothesis tests were used to estimate statistical significance for all categorical comparisons. The prevalence of psychological distress was estimated as a dichotomized variable (a score of ≥4 representing distress – a standardised cut-off).[26] To explore the relationship between LBP and physical and mental health, unconditional logistic regression analyses were carried out. Univariable logistic regression was used to summarise the relationship between (i) LBP and self-rated health, and (ii) LBP and Psychological distress. Multivariable logistic regression was carried out adjusting for age, sex, educational level, and socio-economic status. Comparisons of these associations between settings were illustrated using high-low-close charts. All analyses were done with SPSS version 22, Microsoft Excel and Microsoft PowerPoint version 2013.

Patient and Public Involvement

Being a secondary analysis of an already existing dataset, the public and patients were not involved in the design of this study.

For peer review only - http://bmjopen.bmj.com/site/about/guidelines.xhtml

RESULTS

Distribution of Low back pain by place and person.

The one-month period prevalence of LBP in Europe was 44.6% (95% CI: 43.87 – 45.33). The prevalence ranged from 33.4% (95% CI: 30.21 – 36.59) in Norway to 67.7% (95% CI: 65.26 – 70.14) in Lithuania (figure 1.0). The prevalence in Lithuania, Slovenia, Germany, Slovakia, and Romania were all significantly higher than the overall European prevalence. As shown also, the prevalence of LBP in the UK, Macedonia, The Netherlands, and Norway were all significantly lower than the overall prevalence. Although the prevalence in France was lower than the overall prevalence, it did not achieve statistical significance.

Prevalence varied widely across the EURO URHIS 2 cities (table A supplementary material). Approximately two out of three adults in eastern European cities such as Siauliai, Kaunas, and Maribor reported LBP whereas just about one in three reported symptoms Stockport, Birmingham, and Skopje. Noticeably, the cities whose LBP prevalence fell below the overall European prevalence were dominated by urban areas within the United Kingdom. Of all UK cities included in the survey, only Knowsley had a prevalence rate greater than the overall prevalence.

Prevalence was significantly higher for women (48.2%) than for men (41%) (table B supplementary material). The same pattern was consistent across most of the EURO URHIS 2 countries the only exception being Macedonia. Although male prevalence was higher in Macedonia, this was not significant. Female prevalence in Romania, Turkey, UK, and the Netherlands were statistically significantly higher than those of their male counterparts. The male to female gap was widest in Turkey.

For peer review only - http://bmjopen.bmj.com/site/about/guidelines.xhtml

Generally, (as shown in table c supplementary material) the prevalence of LBP significantly increased with age. The 18 – 39 age group had a prevalence of 34.8%, whilst the 40 – 59 age group had 46.3%. The highest prevalence of 48.2% was recorded by over 60s. This pattern was found to be fairly consistent across all the participating countries the only exception being Germany. Furthermore, the 18-39 age group across most of the countries recorded similar prevalence rates which was around ~30%. In the UK, the Netherlands and Norway, none of the age groups recorded prevalence rates of up to 40%. The highest differential in prevalence with respect to age was found in Romania where the prevalence doubles between the lowest and highest age categories.

On the whole, it can be seen from table 1.0 that people in the 40-59 age group had a 61% higher chance of LBP (95% CI: 1.48 – 1.76) than people in the 18-39 age group. Similarly, the 60 and above age group had the highest unadjusted odds for LBP which was about 74% increased odds (95% CI: 1.61 – 1.88) compared to the youngest group. The one-month period prevalence of LBP in the study was found to vary significantly with a generic measure of socio-economic status and individuals' level of education. Participants who reported not being able to meet up with their daily expenses had an almost two-fold increase in risk of LBP compared to those who could (Odds Ratio [OR]: 1.96, 95% CI: 1.83 – 2.11). Persons with just primary or secondary education had more than a two-fold increase in the risk of LBP compared to those who had university education. Whereas those with No formal education had a 44% increase in odds (95% CI: 1.35 – 1.55).

For peer review only - http://bmjopen.bmj.com/site/about/guidelines.xhtml

Table 1.0 Unadjusted Odds ratios of LBP and associated socio-demographic variables.

	Experienced Low back pain in the last one month.			
	N	%	OR	95% CI
**Sex**				
Male	8774	41	Ref	-
Female	8801	48.2	1.34	1.26 – 1.42
**Age**				
18-39	4022	34.8	Ref	-
40-59	4808	46.3	1.61	1.48 – 1.76
≥ 60	8745	48.2	1.74	1.61 – 1.88
**SES**				
Able to meet daily expenses	12,569	40.6	Ref	-
Not able to	4064	57.3	1.96	1.83 – 2.11
**Education**				
University education	5721	37.0	Ref	-
Secondary education	9200	45.9	2.24	1.85 – 2.72
Primary education	1904	57.2	2.28	2.05 – 2.54
No formal education	468	56.8	1.45	1.35 – 1.55

Associated physical and mental health burden of LBP.

This study found almost a four-fold increased risk of having 'less than good' physical health in LBP sufferers compared to those who did not have LBP (shown in table 2.0). After accounting for the effects of age, sex, socio-economic status and level of education, only a mild attenuation

For peer review only - http://bmjopen.bmj.com/site/about/guidelines.xhtml

was noticed. The adjusted Odds ratio for the association was 3.54 (95% CI: 3.31 – 3.80; p-value = .0001).

Table 2.0: Association between LBP and self-rated health and psychological distress.

		N	%	OR	95% CI	p
	‘Less than good’ self-rated health					
Low back pain	No	3,076	39.3	Ref	-	-
	Yes (unadjusted)	4,759	60.7	3.95	3.71 – 4.21	.0001
	Yes* (adjusted)			3.54	3.31 – 3.80	.0001
	Psychological Distress					
Low back Pain	No	2,267	49	Ref	-	-
	Yes (unadjusted)	2,355	51	1.64	1.50 – 1.78	.0001
	Yes* (adjusted)			1.44	1.32 – 1.58	.0001

*Adjusted for sex, age, socio-economic status and educational level

This association between LBP and poor self-rated physical health was consistent across all participating countries with OR ranging from 2.14 (95% CI: 1.77-3.28) in France to 4.30 (95% CI: 3.19-5.79) in Lithuania (shown in Figure 2.0). This association is strongest in the eastern European countries of Lithuania and Macedonia as well as in Germany.

There was also strong association between LBP and Psychological distress with OR of 1.64 (95% CI: 1.50 – 1.78), indicating a 64% increase in odds of Psychological distress associated with the occurrence of LBP. As shown in Table 2.0. above, after adjusting for the effects of age, sex, socio-economic status and level of education, the risk was slightly attenuated with a 44%

For peer review only - http://bmjopen.bmj.com/site/about/guidelines.xhtml

elevated risk of PD amongst sufferers of LBP when compared to non-sufferers (95% CI: 1.32 – 1.58; p-value = .0001).

As can be seen in figure 3.0, the association between LBP and psychological distress varies widely between countries. It ranges from being non-existent in Macedonia and Norway to statistically significant association in the Netherlands, UK, Romania, Germany, and Lithuania as indicated by their ORs and associated confidence intervals. In the latter countries, those suffering LBP had between 39% (95% CI: 1.012 – 1.976) to 104% (95% CI: 1.4771 – 2.841) greater odds of psychological distress compared to non-sufferers. For Turkey, France, Slovenia, and Slovakia, there was a slight increase in odds of psychological distress amongst those suffering from LBP though not statistically significant.

Similarly, figure 4.0 shows that the association of LBP and psychological distress across the various urban areas varies widely from and OR of 0.78 (95% CI: 0.50 – 1.21) in Skopje to 2.56 (95% CI: 1.43 – 3.57) in Siauliai.

DISCUSSION

This research found that the one-month period prevalence of low back pain (LBP) in Europe was 44.6% widely ranging between countries from 33.4% - 67.7%. This finding aligns with findings from previous research assessing one-month period prevalence carried out among European populations.[27-29] A Greek cross-sectional study[29] found a prevalence of 37.1% whilst a cohort study carried out in the north-west of England reported a 39% prevalence.[27] Furthermore, a series of five cross sectional surveys conducted over a 20-year period in Finland with a cumulative response of 29,043 revealed an adult prevalence ranging between 46%-

For peer review only - http://bmjopen.bmj.com/site/about/guidelines.xhtml

51%.[28] A similar one-month period prevalence was found by a recent systematic review.[30] However, these earlier research works were limited due to lack of comparability across heterogeneous settings. This study demonstrated a wide variation in LBP prevalence across European urban areas. Considering that data on LBP was collected using a previously validated question with an optimal recall period, and that the same instrument was used in all participating cities, the differences are most likely to be real. Additionally, to minimize the likelihood of information bias resulting from confusions of language or other cultural factors, all questionnaires were translated and back-translated for the survey. LBP is usually managed at local general practitioners' clinics hence it is plausible that places with easier access to health services might have lower prevalence as the sufferers may receive early medical attention. This relatively high prevalence has implications for healthcare resource allocation as cities with higher proportion of their adult population suffering LBP should be expected to allocate more resources towards its care.

This research also found that the female sex, older people and people of lower socio-economic status as well as the less educated are at greatest risk of LBP (table 1.0). Although similar associations with lower socio-economic status, less education, and older age have been consistent from earlier studies,[31,32] opinion is divided on the sex differences. A notable review done for the global burden of disease study found greater prevalence in males across all regions of the world.[1] However, the studies that specifically assessed one-month period prevalence found higher proportions amongst females.[27-29]

Irrespective of sex, age, socio-economic status or level of education, this study found that adult Europeans suffering from LBP have three-and-half-fold higher odds of experiencing a poor state of physical health when compared to non-sufferers. This association is stronger than findings

For peer review only - http://bmjopen.bmj.com/site/about/guidelines.xhtml

from earlier studies amongst European populations. A Finnish cross sectional study reported an association between chronic pain and ‘poor self-rated health’ with OR ranging from 1.16 – 2.62 depending on the frequency of the pain.[33] On the other hand, a British cohort study found that background self-rated poor health is a predictor of chronicity amongst LBP users of primary care.[34] Although an OR of 3.6 (95% CI: 1.9 – 6.8) was found, estimates had wide confidence intervals as the sample size was only 180. However, whether as predictor or consequence, no previous study has compared the strength of this association across different European settings. Being a cross-sectional survey, this association, though strong, consistent across all cities and countries, and highly significant, cannot be assumed to be causal. It is also noteworthy to observe that the pattern of this association was similar to the pattern of the prevalence of LBP across countries. Implying that countries with relatively higher prevalence of LBP also tended to have stronger association with poor physical health (figure 2.0).

Another key finding of this research is that adult urban Europeans with LBP stand a 44% higher risk of psychological distress. An American longitudinal study similarly found that LBP at baseline was associated with a higher risk of psychological distress at the 18th month review with an OR of 1.36 (95% CI: 1.07 – 1.72).[35] The research had a much smaller sample size of 681 and was restricted to only the US. But why is this association important? It is known that amongst sufferers of LBP, psychological distress predicts response to therapy and chronicity both of which have significant resource implications.[10,13] It also predicts occurrence of disability[11] and thus predicts poverty given that Europeans living with a disability are significantly poorer than those who are not.[36] This finding has potentially far reaching implications for planning public health interventions, resource allocation, and social services planning and delivery. More so in countries like the UK, and the Netherlands who despite having

For peer review only - http://bmjopen.bmj.com/site/about/guidelines.xhtml

a comparatively low prevalence of LBP (figure 1.0), have significantly higher burdens of its associated poor mental health (figure 3.0). The same goes for urban areas such as Wirral, Craiova, and Tameside and Glossop three of which have relatively low LBP prevalence but higher burdens of psychological distress associated LBP (figure 4.0).

Furthermore, the distribution of LBP in urban Europe varied differently from the distribution of its associated mental health burden. Implying that prevalence figures alone do not represent the associated mental health burden of LBP and likewise may not represent its other associated burdens and impact. Therefore, health policy makers will benefit greatly from understanding the associated burdens of LBP in the context of particular urban areas to help fashion appropriate interventions amongst high-risk and general populations.

Globally, Low back pain is quite prevalent in high income countries and in low-and-middle income countries.[37] However, within the relatively affluent European countries, this study shows that it is the female sex, the older people, the less educated and the poorer people that are more likely to be affected by it. Low back pain has thus become an embodiment of engrained social mechanisms that pattern health and determinants of health in urban European societies. It has long been held that these mechanisms are not inevitable but remediable.[38] Governments at national and city levels are thus expected to make policies that will protect the vulnerable members of society from the worst effects of LBP.

Expectedly, the associated burden of LBP on physical health travels in much the same direction as its prevalence. However, its associated mental health burdens are a bit subtler. Areas of relatively low LBP prevalence may have a greater associated psychological distress. The factors that shape these differences may be connected to ease of access to care, as well as availability of support to persons and families affected by LBP. This again, may be a pointer to underlying

For peer review only - http://bmjopen.bmj.com/site/about/guidelines.xhtml

intersection of factors prevalent in those urban settings. Much as such factors deserve further exploration through research, the findings thus far should inform public health policy and action as well as the (re)structuring of social services. LBP associated with psychological distress is more likely to result in disability and eventually, poverty. Added to the demands of seeking and accessing care, families run a higher risk of being driven into further impoverishment. This potentially widens the inequalities gap in societies creating a vicious circle of disease, disability and disadvantage.

The data informing this analysis was generated from a population cross-sectional study. As a result, the temporal relationship between exposure and outcomes cannot be established by our research as both information were collected at the same time. Despite rigorous efforts to minimize bias in the EURO URHIS 2 study, such surveys may retain the possibility of information bias on account of poor recall as well as selection bias from non-response. Additionally, our research is unable to explore if a dose-response relationship exists between LBP and psychological distress. Further research is required to answer these important questions. We also note that there was no pre-specified analysis plan in the protocol for the particular variables presented in this paper.

ACKNOWLEDGEMENTS

The authors acknowledge all the members of the European Urban Health Indicators Survey 2 research team.

FUNDING STATEMENT

For peer review only

This work was supported by [The Commonwealth Scholarship Commission] grant number [NGSS-184-2015]

COMPETING INTERESTS

The authors have no competing interests to declare.

CONTRIBUTORSHIP STATEMENT

CIU and DP contributed to the conceptualization and the design of this study, DP facilitated the acquisition of the data, CIU and DP contributed to the analysis and interpretation of the data, both authors contributed to the drafting and proofreading of the manuscripts and have approved of its submission.

DATA SHARING

The full dataset associated with this study is held by the EURO URHIS 2 study team and can be made available upon reasonable request by an email to: danpope@liverpool.ac.uk

ETHICS STATEMENT

Not applicable/No human participants included.

For peer review only

For peer review only - http://bmjopen.bmj.com/site/about/guidelines.xhtml

REFERENCES

1 Hoy D, March L, Brooks P, et al. The global burden of low back pain: estimates from the Global Burden of Disease 2010 study. *Ann Rheum Dis* 2014 Jun;73(6):968-974.

2 Murray CJ, Vos T, Lozano R, et al. Disability-adjusted life years (DALYs) for 291 diseases and injuries in 21 regions, 1990–2010: a systematic analysis for the Global Burden of Disease Study 2010. *The lancet* 2013;380(9859):2197-2223.

3 Croft PR, Macfarlane GJ, Papageorgiou AC, et al. Outcome of low back pain in general practice: a prospective study. *Br Med J* 1998 May 2;316(7141):1356-1359.

4 Dagenais S, Caro J, Haldeman S. A systematic review of low back pain cost of illness studies in the United States and internationally. *The Spine journal* 2008;8(1):8-20.

5 Maetzel A, Li L. The economic burden of low back pain: a review of studies published between 1996 and 2001. *Best Practice & Research Clinical Rheumatology* 2002;16(1):23-30.

6 Lewandowski W, Morris R, Draucker CB, et al. Chronic pain and the family: Theory-driven treatment approaches. *Issues Ment Health Nurs* 2007;28(9):1019-1044.

7 Kovacs FM, Abraira V, Zamora J, et al. Correlation between pain, disability, and quality of life in patients with common low back pain. *Spine* (Phila Pa 1976) 2004 Jan 15;29(2):206-210.

8 Koes B, Van Tulder M, Thomas S. Diagnosis and treatment of low back pain. *Br Med J* 2006;7555:1430.

9 Van Tulder MW, Scholten RJ, Koes BW, et al. Nonsteroidal anti-inflammatory drugs for low back pain: a systematic review within the framework of the Cochrane Collaboration Back Review Group. *Spine* 2000;25(19):2501-2513.

10 Gurung T, Ellard DR, Mistry D, et al. Identifying potential moderators for response to treatment in low back pain: A systematic review. *Physiotherapy* 2015;101(3):243-251.

11 Lee H, Hubscher M, Moseley GL, et al. How does pain lead to disability? A systematic review and meta-analysis of mediation studies in people with back and neck pain. *Pain* 2015 Jun;156(6):988-997.

12 Pincus T, Vlaeyen JW, Kendall NA, et al. Cognitive-behavioral therapy and psychosocial factors in low back pain: directions for the future. *Spine* (Phila Pa 1976) 2002 Mar 1;27(5):E133-8.

13 Pincus T, Burton AK, Vogel S, et al. A systematic review of psychological factors as predictors of chronicity/disability in prospective cohorts of low back pain. *Spine* 2002;27(5):109-120.

For peer review only - http://bmjopen.bmj.com/site/about/guidelines.xhtml

14 Vargas-Prada S, Serra C, Martinez JM, et al. Psychological and culturally-influenced risk factors for the incidence and persistence of low back pain and associated disability in Spanish workers: findings from the CUPID study. *Occup Environ Med* 2013 Jan;70(1):57-62.

15 Gunzburg R. Low back pain: in search of goddess Panacea. *Eur Spine J* 2014;23(10):2027.

16 Verma A, van Ameijden E, Birt C, et al. EURO-URHIS 1 and 2 consortia. Why investigate urban health indicators? *Eur J Public Health* 2015 Jul 21.

17 Economic and Social Affairs, United Nations. World Urbanization Prospects The 2007 Revision Highlights. 2008(1):230.

18 Pope D, Puzzolo E, Birt C, et al. Collecting standardised urban health indicator data at an individual level for adults living in urban areas: methodology from EURO-URHIS 2. *Eur J Public Health* 2016 Jan 7.

19 Higgerson J, Birt C, van Ameijden E, et al. Defining the urban area for cross national comparison of health indicators: the EURO-URHIS 2 boundary study. *Eur J Public Health* 2015 Jul 23.

20 Dionne CE, Dunn KM, Croft PR, et al. A consensus approach toward the standardization of back pain definitions for use in prevalence studies. *Spine* (Phila Pa 1976) 2008 Jan 1;33(1):95-103.

21 Goldberg D, Williams P. User's Guide to the GHQ. Windsor, NFER Nelson 1988.

22 Pevalin DJ. Multiple applications of the GHQ-12 in a general population sample: an investigation of long-term retest effects. *Soc Psychiatr Epidemiol* 2000;35(11):508-512.

23 Idler EL, Benyamini Y. Self-rated health and mortality: a review of twenty-seven community studies. *J Health Soc Behav* 1997:21-37.

24 Bowling A. Just one question: If one question works, why ask several? *J Epidemiol Community Health* 2005 May;59(5):342-345.

25 Eriksson I, Unden AL, Elofsson S. Self-rated health. Comparisons between three different measures. Results from a population study. *Int J Epidemiol* 2001 Apr;30(2):326-333.

26 Lundin A, Åhs J, Åsbring N, Kosidou K, Dal H, Tinghög P, Saboonchi F, Dalman C. Discriminant validity of the 12-item version of the general health questionnaire in a Swedish case-control study. Nordic journal of psychiatry. 2017 Apr 3;71(3):171-9.

27 Croft PR, Papageorgiou AC, Ferry S, et al. Psychologic distress and low back pain: evidence from a prospective study in the general population. *Spine* 1995;20(24):2731-2737.

For peer review only - http://bmjopen.bmj.com/site/about/guidelines.xhtml

28 Heistaro S, Vartiainen E, Heliovaara M, et al. Trends of back pain in eastern Finland, 1972-1992, in relation to socioeconomic status and behavioral risk factors. *Am J Epidemiol* 1998 Oct 1;148(7):671-682.

29 Stranjalis G, Tsamandouraki K, Sakas DE, et al. Low back pain in a representative sample of Greek population: analysis according to personal and socioeconomic characteristics. *Spine* 2004;29(12):1355-1360.

30 Hoy D, Bain C, Williams G, et al. A systematic review of the global prevalence of low back pain. *Arthritis & Rheumatism* 2012;64(6):2028-2037.

31 Manek NJ, MacGregor AJ. Epidemiology of back disorders: prevalence, risk factors, and prognosis. *Curr Opin Rheumatol* 2005 Mar;17(2):134-140.

32 Hoy D, Brooks P, Blyth F, et al. The epidemiology of low back pain. *Best practice & research Clinical rheumatology* 2010;24(6):769-781.

33 Mäntyselkä PT, Turunen JH, Ahonen RS, et al. Chronic pain and poor self-rated health. *JAMA* 2003;290(18):2435-2442.

34 Thomas E, Silman AJ, Croft PR, et al. Predicting who develops chronic low back pain in primary care: a prospective study. *Br Med J* 1999 Jun 19;318(7199):1662-1667.

35 Hurwitz EL, Morgenstern H, Yu F. Cross-sectional and longitudinal associations of low-back pain and related disability with psychological distress among patients enrolled in the UCLA Low-Back Pain Study. *J Clin Epidemiol* 2003;56(5):463-471.

36 Hosking D. Staying the Course: The European Disability Strategy 2010–2020. *European yearbook of disability law* 2013;4:73-99.

37 Morris LD, Daniels KJ, Ganguli B, Louw QA. An update on the prevalence of low back pain in Africa: a systematic review and meta-analyses. BMC Musculoskeletal Disorders. 2018 Dec;19(1):1-5.

38 Whitehead M, Dahlgren G, World Health Organization. Levelling up (part 1): a discussion paper on concepts and principles for tackling social inequities in health. 2006.

For peer review only

Figure 1.0 Prevalence of LBP with 95% CI in the EURO URHIS 2 Countries.

Figure 2.0 Association between LBP and self-rated ‘less than good’ health.

Figure 3.0 Association between LBP and Psychological Distress in the EURO URHIS 2 Countries.

Figure 4.0 Association between LBP and Psychological Distress in the EURO URHIS 2 cities.

For peer review only

For peer review only - http://bmjopen.bmj.com/site/about/guidelines.xhtml

Figure 1.0

Figure 1.0 Prevalence of LBP with 95% CI in the EURO URHIS 2 Countries.

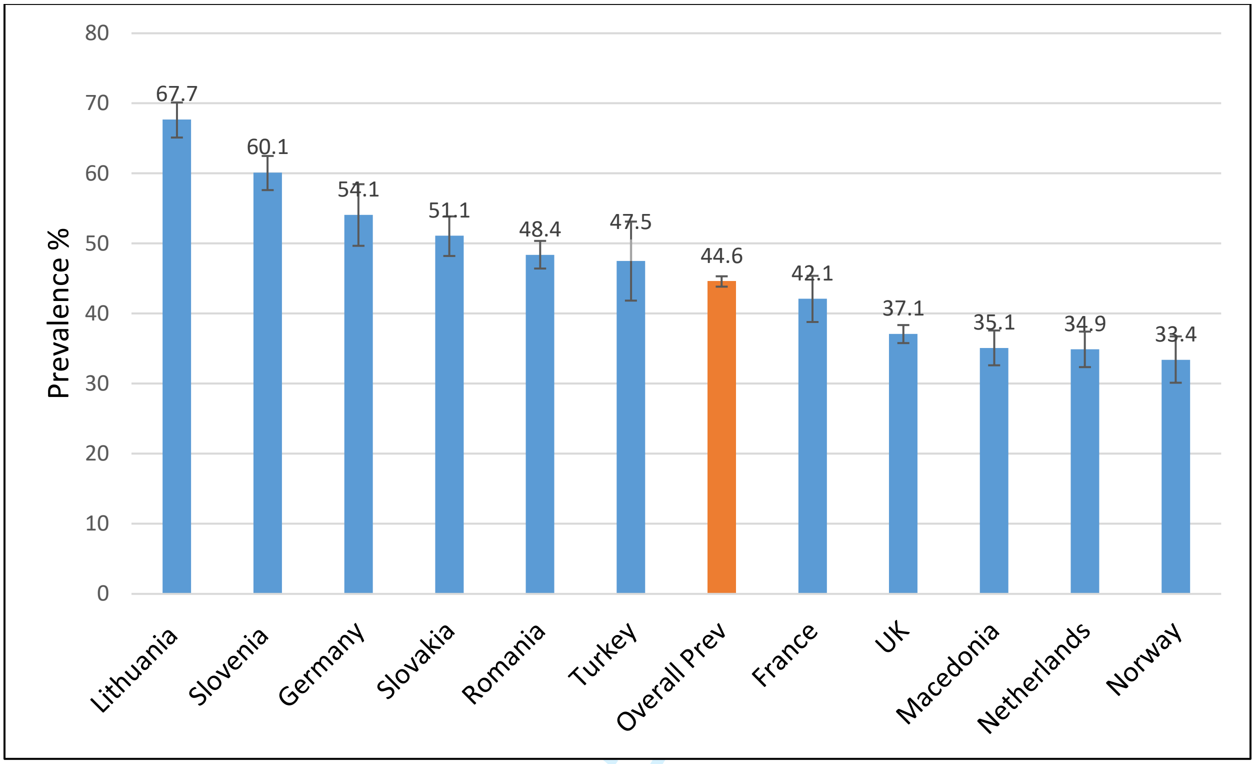

For peer review only

Figure 2.0

Figure 2.0: Association between LBP and self-rated 'less than good' health

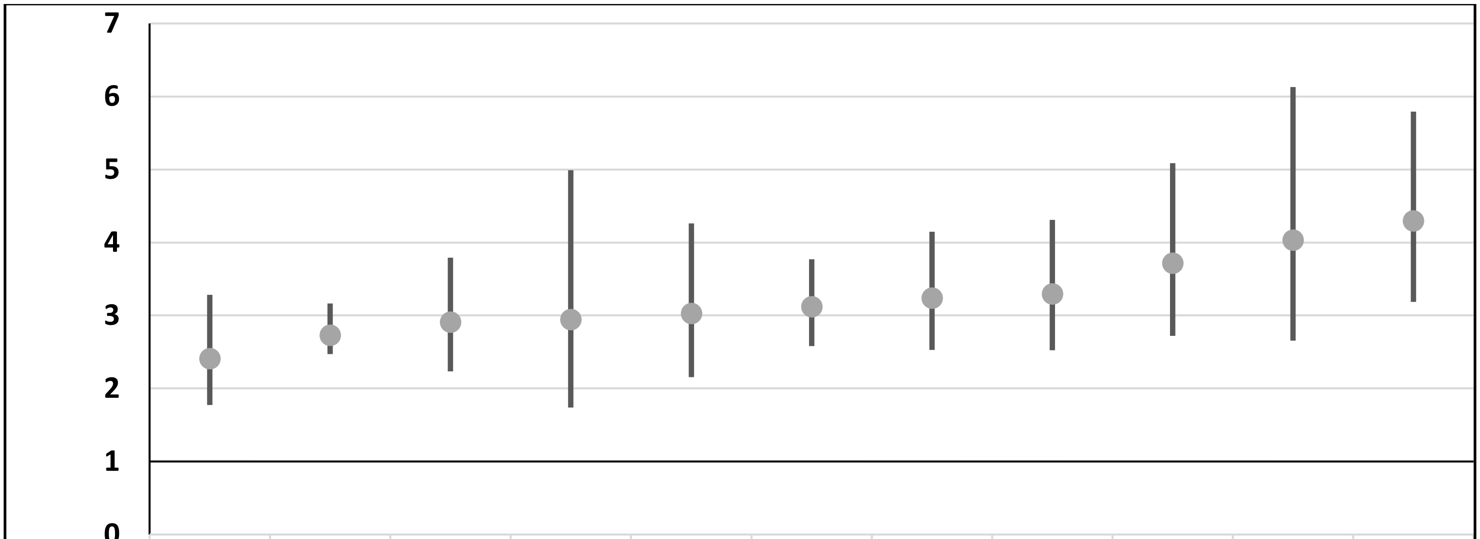

	France	UK	Netherlands	Turkey	Norway	Romania	Slovenia	Slovakia	Macedonia	Germany	Lithuania
High	3.281	3.166	3.793	4.988	4.261	3.769	4.148	4.309	5.084	6.129	5.794
Low	1.773	2.47	2.236	1.739	2.154	2.581	2.53	2.524	2.721	2.655	3.188
● Close	2.412	2.729	2.912	2.945	3.03	3.119	3.239	3.298	3.719	4.034	4.298

High = Upper 95% Confidence Interval, Low = Lower 95% confidence Interval

• Close = Adjusted Odds ratio of the Association between Low back pain and Psychological distress (adjusted for sex, age socio-economic status, and educational level)

review only

Figure 3.0

Figure 3.0: Association between LBP and Psychological Distress in the EURO URHIS 2 Countries

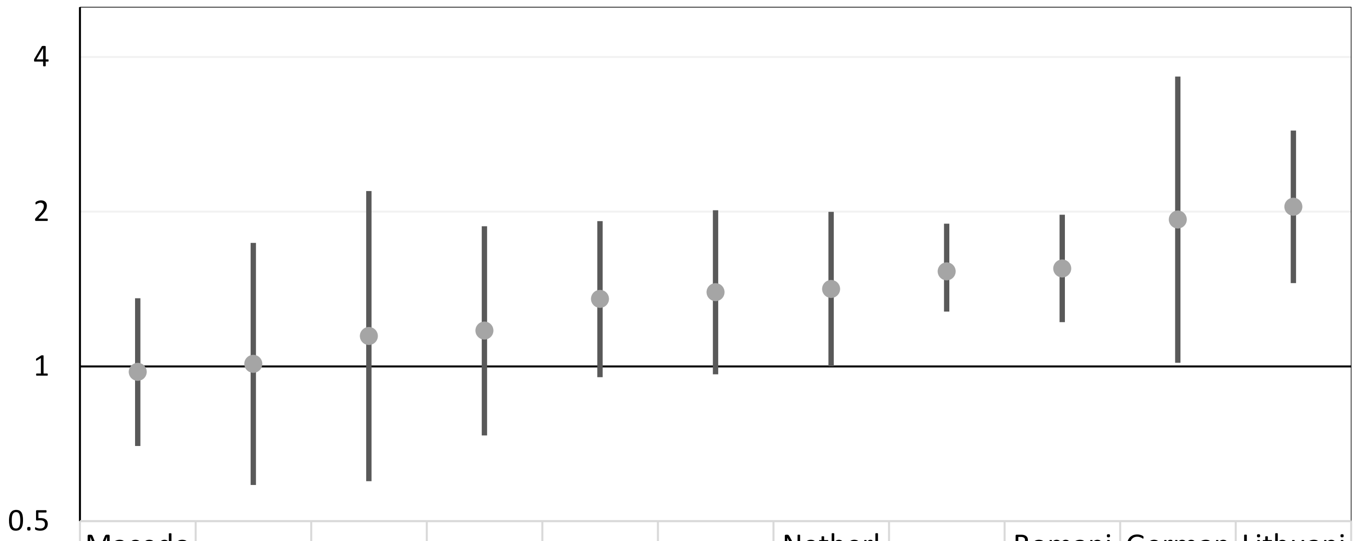

	Macedonia	Norway	Turkey	France	Slovenia	Slovakia	Netherlands	UK	Romania	Germany	Lithuania
High	1.342	1.718	2.167	1.851	1.895	1.99	1.976	1.872	1.949	3.621	2.841
Low	0.709	0.595	0.605	0.743	0.965	0.977	1.012	1.293	1.234	1.029	1.471
● Close	0.975	1.011	1.145	1.173	1.352	1.394	1.414	1.53	1.551	1.93	2.044

High = Upper 95% Confidence interval; Low = Lower 95% Confidence Interval

• Close = Adjusted Odds ratio of the association between low back pain and Psychological distress (adjusted for sex, age socio-economic status, and educational level)

For peer review only - http://bmjopen.bmj.com/site/about/guidelines.xhtml

Figure 4.0

Figure 4.0: Association between LBP and Psychological Distress in the EURO URHIS 2 cities

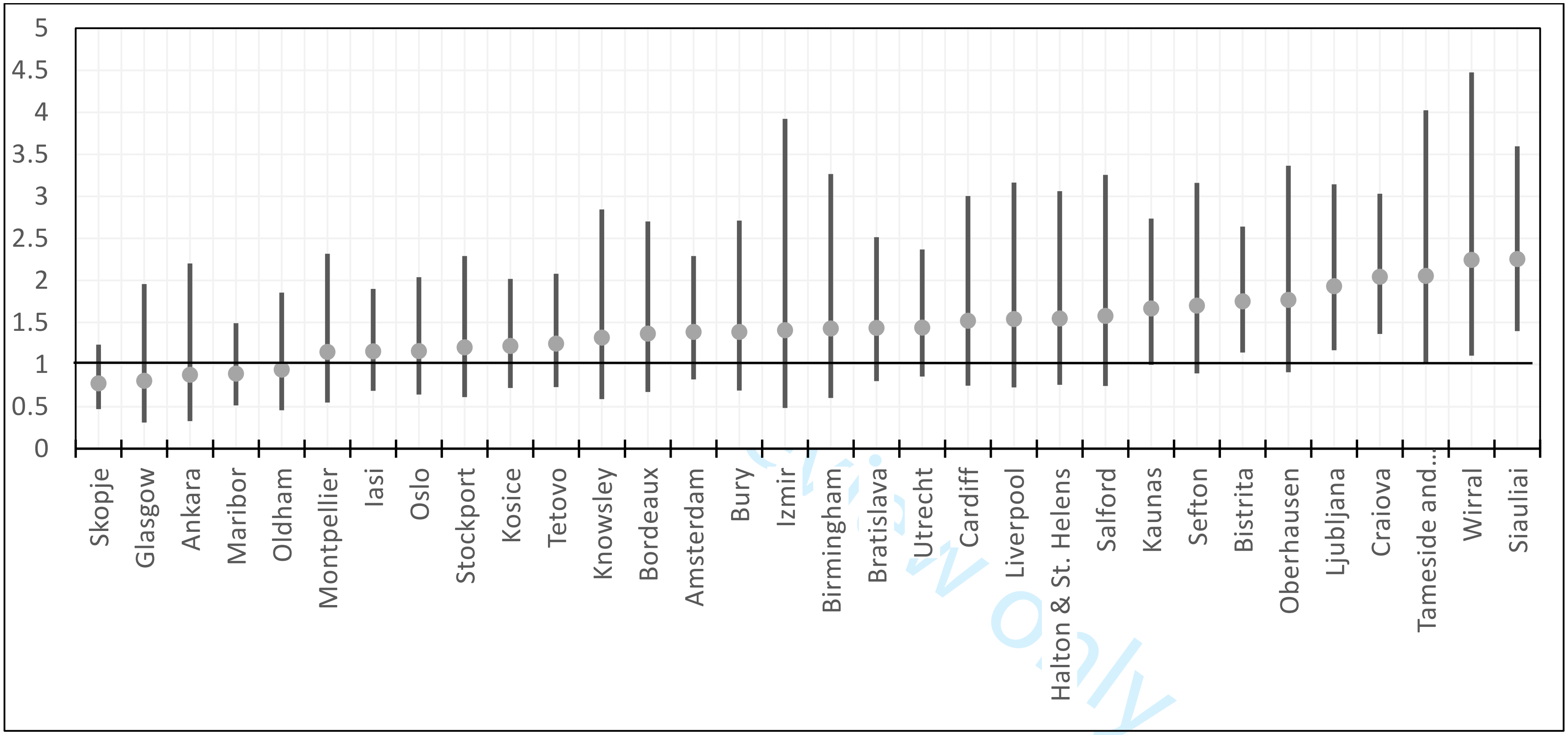

Adjusted Odds ratio of the Association between Low back pain and Psychological distress (adjusted for sex, age socio-economic status, and educational level)

Table A Supplementary Material

Prevalence of LBP in the EURO URHIS 2 UAs with 95% CIs

		Experienced LBP in the past one month			
Urban Area	N	%	95% CI		
			Lower CI	Upper CI	
Skopje	764	31.3	28.01	34.59	Macedonia
Birmingham	291	31.6	26.2	36.94	United Kingdom
Stockport	534	32.2	28.24	36.16	United Kingdom
Oslo	809	33.4	30.15	36.65	Norway
Utrecht	731	34.1	30.66	37.54	The Netherlands
Wirral	395	34.4	29.72	39.08	United Kingdom
Salford	399	35.6	30.9	40.3	United Kingdom
Amsterdam	663	35.7	32.05	39.35	The Netherlands
Cardiff	437	35.7	31.21	40.19	United Kingdom
Halton & St. Helens	384	36.5	31.68	41.32	United Kingdom
Bury	443	36.8	32.31	41.29	United Kingdom
Liverpool	440	37.5	32.98	42.02	United Kingdom
Tameside and Glossop	472	37.9	33.52	42.28	United Kingdom
Sefton	557	37.9	33.87	41.93	United Kingdom
Craiova	848	38	34.73	41.27	Romania
Oldham	437	38.9	34.33	43.47	United Kingdom
Tetovo	693	39.2	35.57	42.83	Macedonia
Glasgow	270	40.7	34.84	46.56	United Kingdom
Bordeaux	443	41.1	36.52	45.68	France
Montpellier	446	43	38.41	47.59	France
Izmir	136	46.3	37.92	54.68	Turkey
Knowsley	412	46.6	41.78	51.42	United Kingdom
Ankara	180	48.3	41	55.6	Turkey
Kosice	689	48.8	45.07	52.53	Slovakia
Bistrita	926	51.9	48.68	55.12	Romania
Bratislava	547	53.9	49.72	58.08	Slovakia
Oberhausen	514	54.1	49.79	58.41	Germany
Ljubljana	775	55.6	52.1	59.1	Slovenia
Iasi	751	55.8	52.25	59.35	Romania
Maribor	813	64.3	61.01	67.59	Slovenia
Kaunas	691	67.6	64.11	71.09	Lithuania
Siauliai	685	67.7	64.2	71.2	Lithuania

For peer review only

Table B Supplementary Material

Adult Male/Female Prevalence of Low back pain in the EURO URHIS 2 Study.

Country			Total	Low back pain<br>Percentage with LBP	95% CI<br>Lower CI	Upper CI	P value<br>p
UK	Sex	Male	2666	34.7	32.89	36.51	.0001
		Female	2805	39.3	37.49	41.11	
Netherlands		Male	654	30.4	26.87	33.93	.001
		Female	740	38.8	35.29	42.31	
Turkey		Male	179	39.7	32.53	46.87	.001
		Female	137	57.7	49.43	65.97	
Romania		Male	1137	41.6	38.74	44.46	.0001
		Female	1388	54.0	51.38	56.62	
France		Male	429	39.6	34.97	44.23	.154
		Female	460	44.3	39.76	48.84	
Slovakia		Male	598	47.8	43.80	51.80	.028
		Female	638	54.1	50.23	57.97	
Lithuania		Male	595	65.0	61.97	68.83	.07
		Female	781	69.7	66.48	72.92	
Slovenia		Male	785	58.3	54.85	61.75	.164
		female	803	61.8	58.44	65.16	
Germany		Male	233	50.6	44.18	57.02	.154
		female	281	56.9	51.11	62.69	
Norway		Male	414	30.2	25.78	34.62	.049
		Female	395	36.7	31.95	41.45	
Macedonia		Male	1084	35.3	32.46	38.14	.723
		Female	373	34.3	29.48	39.12	
Europe Total		Male	8774	41.0	39.97	42.03	.0001
		Female	8801	48.2	47.16	49.24	
**Total**			17575	44.6	43.87	45.33	

Table C Supplementary Material

Adult age prevalence of LBP in the EURO URHIS 2 Study

			Experienced LBP in the past month			
			%	95% CI		P
	Age	N		Lower CI	Upper CI	
Lithuania	18-39	301	56.5	50.90	62.10	.0001
	40-59	396	68.4	63.82	72.98	
	60+	679	72.2	68.83	75.57	
Slovenia	18-39	298	46.6	40.94	52.26	.0001
	40-59	429	60.6	55.98	65.22	
	60+	861	64.5	61.30	67.77	
Germany	18-39	79	41.8	30.92	52.68	.039
	40-59	138	59.4	51.21	67.59	
	60+	297	54.9	49.24	60.56	
Slovakia	18-39	198	33.3	26.74	39.86	.0001
	40-59	313	48.2	42.66	53.74	
	60+	725	57.1	53.50	60.70	
Romania	18-39	877	32.3	29.21	35.39	.0001
	40-59	953	51.5	48.33	54.67	
	60+	695	64.5	60.94	68.06	
Turkey	18-39	88	47.7	37.26	58.14	.936
	40-59	140	46.4	38.14	54.66	
	60+	88	48.9	38.46	59.34	
France	18-39	114	33.3	24.65	41.95	.022
	40-59	294	47.6	41.89	53.31	
	60+	481	40.7	36.31	45.09	
UK	18-39	855	31.0	27.90	34.10	.0001
	40-59	1372	37.9	35.33	40.47	
	60+	3244	38.3	36.63	39.97	
Macedonia	18-39	680	30.0	26.56	33.44	.0001
	40-59	351	28.5	23.78	33.22	
	60+	426	48.6	43.85	53.35	
Netherlands	18-39	341	29.9	25.04	34.76	.081
	40-59	268	35.4	29.67	41.13	
	60+	785	36.8	33.43	40.17	
Norway	18-39	191	30.4	23.88	36.92	.452
	40-59	154	31.8	24.44	39.16	
	60+	464	35.1	30.76	39.44	

For peer review only

STROBE Statement—Checklist of items that should be included in reports of ***cross-sectional studies***

	Item No	Recommendation
**Title and abstract**	1	(*a*) Indicate the study's design with a commonly used term in the title or the abstract Page 1 lines 2 - 3
		(*b*) Provide in the abstract an informative and balanced summary of what was done and what was found. Page 2 lines 2 – 20, page 3 lines 1 - 6
**Introduction**		
Background/rationale	2	Explain the scientific background and rationale for the investigation being reported Page 4 lines 2 – 22, page 5 lines 1 - 12
Objectives	3	State specific objectives, including any prespecified hypotheses page 5 lines 10-12
**Methods**		
Study design	4	Present key elements of study design early in the paper page 5 line 14 – 22, page 6 lines 1 - 12
Setting	5	Describe the setting, locations, and relevant dates, including periods of recruitment, exposure, follow-up, and data collection page 5 lines 14 – 22, page 6 lines 1 - 12
Participants	6	(*a*) Give the eligibility criteria, and the sources and methods of selection of participants page 6 lines 8 - 12
Variables	7	Clearly define all outcomes, exposures, predictors, potential confounders, and effect modifiers. Give diagnostic criteria, if applicable Page 6 lines 14 - 18
Data sources/ measurement	8*	For each variable of interest, give sources of data and details of methods of assessment (measurement). Describe comparability of assessment methods if there is more than one group Page 6 lines 14 – 23, page 7 lines 1 - 23
Bias	9	Describe any efforts to address potential sources of bias Page 6 lines 10 - 13
Study size	10	Explain how the study size was arrived at Not Applicable as this is a secondary analysis
Quantitative variables	11	Explain how quantitative variables were handled in the analyses. If applicable, describe which groupings were chosen and why Page 6 lines 14 – 23, page 7 lines 1 - 23
Statistical methods	12	(*a*) Describe all statistical methods, including those used to control for confounding
		(*b*) Describe any methods used to examine subgroups and interactions Page 8 lines 4 - 14
		(*c*) Explain how missing data were addressed Not Applicable
		(*d*) If applicable, describe analytical methods taking account of sampling strategy Not Applicable
		(*e*) Describe any sensitivity analyses Not Applicable
**Results**		
Participants	13*	(a) Report numbers of individuals at each stage of study—eg numbers potentially eligible, examined for eligibility, confirmed eligible, included in the study, completing follow-up, and analysed Not Applicable
		(b) Give reasons for non-participation at each stage Not Applicable
		(c) Consider use of a flow diagram Not Applicable
Descriptive data	14*	(a) Give characteristics of study participants (eg demographic, clinical, social) and information on exposures and potential confounders page 9 lines 2 – 22, page 10 lines 1 – 19, page 11 table 1.0
		(b) Indicate number of participants with missing data for each variable of interest Not Applicable

For peer review only

Outcome data	15*	Report numbers of outcome events or summary measures Figure 1.0
Main results	16	(*a*) Give unadjusted estimates and, if applicable, confounder-adjusted estimates and their precision (eg, 95% confidence interval). Make clear which confounders were adjusted for and why they were included Page 12 table 2.0
		(*b*) Report category boundaries when continuous variables were categorized Page 11 table 1.0, page 12 table 2.0
		(*c*) If relevant, consider translating estimates of relative risk into absolute risk for a meaningful time period Not Applicable
Other analyses	17	Report other analyses done—eg analyses of subgroups and interactions, and sensitivity analyses Figures 2.0, 3.0, and 4.0
**Discussion**		
Key results	18	Summarise key results with reference to study objectives Page 13 lines 13 – 22, page 14 lines 1 – 23, and page 15 lines 1 - 22
Limitations	19	Discuss limitations of the study, taking into account sources of potential bias or imprecision. Discuss both direction and magnitude of any potential bias page 17 lines 4 - 10
Interpretation	20	Give a cautious overall interpretation of results considering objectives, limitations, multiplicity of analyses, results from similar studies, and other relevant evidence Page 16 lines 16 - 17
Generalisability	21	Discuss the generalisability (external validity) of the study results Page 16 lines 1 - 17
**Other information**		
Funding	22	Give the source of funding and the role of the funders for the present study and, if applicable, for the original study on which the present article is based page 17 lines 12 - 17

*Give information separately for exposed and unexposed groups.

Note: An Explanation and Elaboration article discusses each checklist item and gives methodological background and published examples of transparent reporting. The STROBE checklist is best used in conjunction with this article (freely available on the Web sites of PLoS Medicine at http://www.plosmedicine.org/, Annals of Internal Medicine at http://www.annals.org/, and Epidemiology at http://www.epidem.com/). Information on the STROBE Initiative is available at www.strobe-statement.org.

For peer review only

For peer review only - http://bmjopen.bmj.com/site/about/guidelines.xhtml
